# Trace removal of benzene vapour using double-walled metal–dipyrazolate frameworks

**DOI:** 10.1038/s41563-022-01237-x

**Published:** 2022-04-28

**Authors:** Tao He, Xiang-Jing Kong, Zhen-Xing Bian, Yong-Zheng Zhang, Guang-Rui Si, Lin-Hua Xie, Xue-Qian Wu, Hongliang Huang, Ze Chang, Xian-He Bu, Michael J. Zaworotko, Zuo-Ren Nie, Jian-Rong Li

**Affiliations:** 1 Beijing Key Laboratory for Green Catalysis and Separation and Department of Environmental Chemical Engineering, Beijing University of Technology, Beijing, China; 2 The Key Laboratory of Advanced Functional Materials, Ministry of Education, Faculty of Materials and Manufacturing, Beijing University of Technology, Beijing, China; 3 College of Chemistry and Chemical Engineering, Qingdao University, Shandong, China; 4 Bernal Institute and Department of Chemical Sciences, University of Limerick, Limerick, Ireland; 5 State Key Laboratory of Separation Membranes and Membrane Processes, Tianjin Polytechnic University, Tianjin, China; 6 School of Materials Science and Engineering and TKL of Metal and Molecule-Based Material Chemistry, Nankai University, Tianjin, China

**Keywords:** Molecular self-assembly, Metal-organic frameworks

## Abstract

In principle, porous physisorbents are attractive candidates for the removal of volatile organic compounds such as benzene by virtue of their low energy for the capture and release of this pollutant. Unfortunately, many physisorbents exhibit weak sorbate–sorbent interactions, resulting in poor selectivity and low uptake when volatile organic compounds are present at trace concentrations. Herein, we report that a family of double-walled metal–dipyrazolate frameworks, BUT-53 to BUT-58, exhibit benzene uptakes at 298 K of 2.47–3.28 mmol g^−1^ at <10 Pa. Breakthrough experiments revealed that BUT-55, a supramolecular isomer of the metal–organic framework Co(BDP) (H_2_BDP = 1,4-di(1*H*-pyrazol-4-yl)benzene), captures trace levels of benzene, producing an air stream with benzene content below acceptable limits. Furthermore, BUT-55 can be regenerated with mild heating. Insight into the performance of BUT-55 comes from the crystal structure of the benzene-loaded phase (C_6_H_6_@BUT-55) and density functional theory calculations, which reveal that C–H···X interactions drive the tight binding of benzene. Our results demonstrate that BUT-55 is a recyclable physisorbent that exhibits high affinity and adsorption capacity towards benzene, making it a candidate for environmental remediation of benzene-contaminated gas mixtures.

## Abstract (web summary)

Volatile organic compounds such as benzene are toxic pollutants that cause health issues even at trace concentrations. Here, a double-walled metal–organic framework is presented that demonstrates high uptake at very low pressures (<10 Pa), allowing the removal of benzene to below acceptable indoor limits.

## Main

Volatile organic compounds (VOCs) such as benzene are a class of toxic pollutants responsible for both indoor and outdoor air pollution that cause environmental and health issues even at trace concentrations^1–4^. Current methods to remove benzene from indoor air include plasma oxidation, photocatalytic oxidation and adsorption by activated carbons^5^. Oxidation is used to degrade benzene, but secondary pollutants (CO, NO_*x*_ and O_3_) are simultaneously generated^5^. Adsorptive removal of VOCs from polluted air and industrial effluent streams by physisorbents is attractive because of their relatively low recycling energy^6–11^. However, the non-covalent binding forces that typically drive physisorption mean that binding tends to be weak for VOCs, especially at low partial pressure^10–12^.

Metal–organic frameworks (MOFs) are of interest for gas storage and separation as they can offer extra-large surface areas and tunable structures^13–16^, and have been studied for the removal of VOCs^17–26^. Indeed, some MOFs outperform commercial sorbents such as activated carbon fibres, activated carbon (AC) and zeolites in benzene adsorption^6,7,21,22,27^. For example, the benzene uptake by MOF-177 (Basolite Z377)^22^ is 16.8 mmol g^−1^ at 293 K and relative pressure (*P*/*P*_0_) of 1 (compared with 5.6 mmol g^−1^ by AC^9^ and 5.8 mmol g^−1^ by graphitized biocarbon^6^). Although there is clear potential for MOFs to capture VOCs for environmental remediation and personal protection, aromatic VOCs such as benzene tend to form weak electrostatic, hydrogen-bonding or coordination-bonding interactions with MOFs, thereby affording poor separation performance. The capacity of MOFs at high pressures (for example,10 kPa) can be high^22,23^, but this is not a good metric to assess the sorption performance of trace VOCs in air. Several MOFs with high benzene capacity have been reported to perform poorly in simulated air purification in breakthrough experiments^21^. Another concern with respect to MOFs is that stability is a prerequisite for practical utility^28–31^, but many MOFs do not possess good thermal or hydrolytic stability^31–35^. In addition, humidity can also reduce sorption performance^21,32,33^. Here, we report a family of stable MOF adsorbents, guided by crystal engineering, that efficiently remove benzene from air even at parts-per-million levels. These metal–dipyrazolate frameworks, designated here as BUT-53 to BUT-58 (BUT = Beijing University of Technology), are constructed from Co^2+^ or Zn^2+^ ions and ditopic pyrazolate (dipyrazolate) ligands and exhibit similar framework structures but different pore size and chemistry.

### Synthesis and crystal structures

**Fig. 1:**
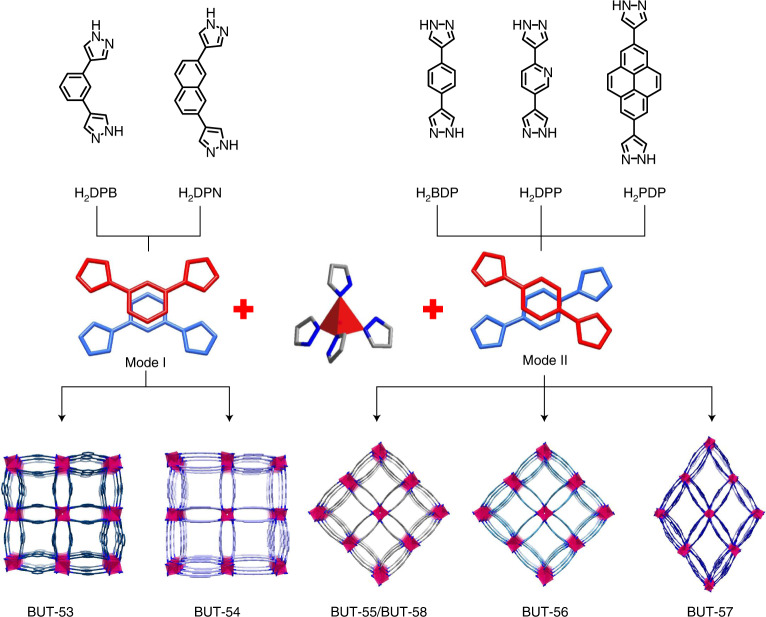
The crystal engineering approach used to design BUT-53 to BUT-58. Schematic representation of the double-walled metal–dipyrazolate frameworks with angular (BUT-53, BUT-54) and linear (BUT-55 to BUT-58) dipyrazolate ligands and Co^2+^- and Zn^2+^-based RBBs.

Solvothermal reactions between dipyrazolate ligands (Fig. 1) and Co^2+^ or Zn^2+^ salts in *N*,*N*-dimethylformamide (DMF) and H_2_O were optimized, in some cases with acetic acid as a modulator, to yield single crystals of BUT-53 to BUT-58 (Supplementary Fig. 1). Single-crystal X-ray diffraction (SCXRD) revealed that BUT-53 and BUT-54 adopt space group *I*4_1_/*amd*, BUT-55, BUT-56 and BUT-58 space group *I*4_1_22, and BUT-57 space group *I*2 (Supplementary Figs. 2–7 and Supplementary Table 1).

**Fig. 2:**
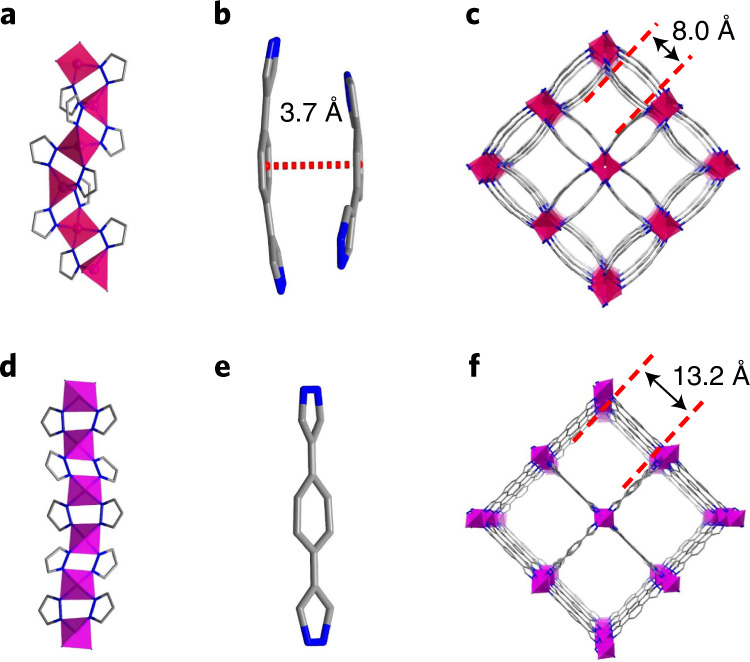
Structural comparison of BUT-55/BUT-58 and Co(BDP)/Zn(BDP). **a**–**c**, Zigzag RBB (**a**), criss-cross stacked BDP^2−^ ligands (**b**) and double-walled 3D framework structure (**c**) of BUT-55 and BUT-58. **d**–**f**, Chain-like RBB (**d**), bridging BDP^2−^ ligand (**e**) and single-walled 3D framework structure (**f**) of Co(BDP) and Zn(BDP).

BUT-55 is formed as a three-dimensional (3D) framework structure comprising Co-based rod building blocks (RBBs) and linear BDP^2−^ (H_2_BDP = 1,4-di(1*H*-pyrazol-4-yl)benzene) linkers (Fig. 1 and Supplementary Fig. 4). The Co^2+^ ions exhibit tetrahedral coordination geometry with pyrazolate (pz) groups from four ligands. Alternating Co^2+^ ions and pz groups generate one-dimensional (1D) RBBs of formula Co(pz)_2_ (Fig. 2a). Each BDP^2−^ ligand links two adjacent Co(pz)_2_ moieties. The central benzene and peripheral pyrazolate rings of BDP^2−^ show some bending (Fig. 2b). Adjacent BDP^2−^ ligands pack in criss-cross mode with the central benzene rings eclipsed, resulting in a double-walled network (Fig. 2c). The term ‘double-walled’ is used here as pairs of pz moieties link adjacent metal cations to form undulating RBBs that are cross-linked by pairs of BDP^2−^ ligands (Fig. 2a–c). This contrasts with the supramolecular isomeric structure^36^ Co(BDP), in which more linear RBBs are cross-linked by BDP^2−^ ligands to form a single-walled arrangement (Fig. 2d–f). BUT-55 exhibits 1D rectangular pores (wall-to-wall distance between ligands ~3.7 Å) and square channels (width ~8.0 Å). These pores, which are key to sorption, lie along the *c* axis (Supplementary Fig. 4). BUT-58 is the Zn^2+^ analogue of BUT-55 (Supplementary Fig. 7).

The use of the BDP^2−^ ligand in MOFs is exemplified by M(BDP) (M = Zn^2+^, Ni^2+^ and Co^2+^), Fe_2_(BDP)_3_ and Ni_8_(OH)_4_(H_2_O)_2_(BDP)_6_ reported by the groups of Long and Navarro^36–39^. Co(BDP) features structural flexibility and methane storage properties^36,40^, and Zn(BDP) is its Zn(II) analogue^39^. BUT-55/BUT-58 and Co(BDP)/Zn(BDP) have the same chemical composition and are supramolecular isomers^41^ but with different pore structure and chemistry as well as sorption performance.

BUT-56 was obtained by replacing the BDP^2−^ ligand in BUT-55 by DPP^2−^ (H_2_DPP = 2,5-di(1*H*-pyrazol-4-yl)pyridine). DPP^2−^ generated pores with a width of 8.0 Å and a wall-to-wall distance between ligands of ~3.8 Å (Supplementary Fig. 5). The longer pyrene-centred PDP^2−^ analogue (H_2_PDP = 2,7-di(1*H*-pyrazol-4-yl)pyrene) afforded BUT-57 with enlarged rhombic pores (width ~11.5 Å and wall-to-wall distance ~3.9 Å; Supplementary Fig. 6). The replacement of the dipyrazolate ligands by the angular variants DPB^2−^ (H_2_DPB = 1,3-di(1*H*-pyrazol-4-yl)benzene) and DPN^2−^ (H_2_DPN = 2,7-di(1H-pyrazol-4-yl)naphthalene) afforded BUT-53 and BUT-54. In contrast to the cross-wise packed ligands in BUT-55 to BUT-58, those in BUT-53 and BUT-54 adopt a nose-to-nose arrangement with the central aromatic rings eclipsed (Fig. 1). BUT-54 has larger pores (width ~10.0 Å and wall-to-wall distance ~3.7 Å; Supplementary Fig. 3) than BUT-53 (width ~7.8 Å and wall-to-wall distance ~3.0 Å; Supplementary Fig. 2) as naphthalene is larger than benzene. Ligand substitution did not change the double-walled framework structure but impacted pore size and chemistry as well as benzene adsorption.

### Porosity and stability

Supplementary Figs. 8a–13a illustrate that the experimental powder X-ray diffraction (PXRD) patterns of as-synthesized BUT-53 to BUT-58 match their simulated patterns calculated from the SCXRD data, indicating bulk purity. The single-walled Co(BDP) and Zn(BDP) were synthesized and analysed in this study for comparison with the BUT MOFs^39,40^. The PXRD patterns of Co(BDP) and Zn(BDP) differ from those of BUT-55 and BUT-58, whicn also support phase purity of the BUT MOFs (Supplementary Figs. 14 and 15). N_2_ adsorption measurements were performed at 77 K to evaluate the porosity of BUT-53 to BUT-58 (Supplementary Fig. 16). Type I adsorption isotherms revealed N_2_ uptakes of 275, 394, 305, 313, 350 and 279 cm^3^ g^−1^ for BUT-53 to BUT-58 at 1 atm, respectively, indicating permanent microporosity. The Brunauer−Emmett−Teller (BET) surface areas determined from the N_2_ adsorption isotherms were 811, 1,128, 873, 897, 970 and 849 m^2^ g^−1^ for BUT-53 to BUT-58, respectively. The pore size distributions, as calculated by density functional theory (DFT), were 8.1, 9.8, 8.4, 8.4, 12.0 and 8.4 Å for BUT-53 to BUT-58, respectively (Supplementary Fig. 17). These results are consistent with the SCXRD structures when van der Waals contacts are considered.

That a MOF must be stable in water, ideally under both acidic and basic aqueous conditions, matters for practical utility. BUT-53 to BUT-58 were thus investigated (Supplementary Figs. 8–13) by treating them with boiling water, aqueous HCl (pH 5) and aqueous NaOH (pH 14) at room temperature for 24 h. BUT-53 to BUT-56 and BUT-58 exhibited phase stability as the PXRD patterns matched their as-synthesized patterns (Supplementary Figs. 8a–11a and 13a). The immersion of BUT-57 in aqueous NaOH (pH 10) for 24 h resulted in reduced PXRD intensity (Supplementary Fig. 12a), implying relatively poor base stability. BUT-53 to BUT-57 retained structural integrity upon exposure to air for 1 year. The N_2_ sorption isotherms of BUT-53 to BUT-58 were also measured after treatment and confirmed that pore collapse/blockage had not occurred (Supplementary Figs. 8b–13b). Thermogravimetric analysis (TGA) of BUT-53 to BUT-58 revealed stability to 408, 363, 371, 387, 395 and 432 °C, respectively (Supplementary Fig. 18).

### Benzene adsorption and capture

**Fig. 3:**
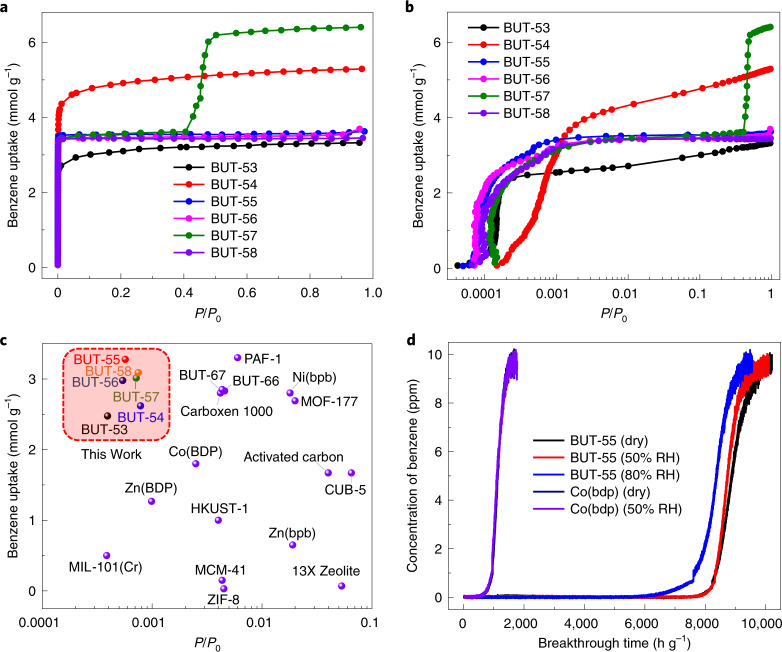
Benzene adsorption studies. a, Benzene adsorption isotherms of BUT-53 to BUT-58 at 298 K. b, Logarithmic-scale plots of *P*/*P*_0_ to view the benzene adsorption of BUT-53 to BUT-58 at low partial pressure. c, Benzene uptakes of BUT-53 to BUT-58 compared with representative porous sorbents at low relative pressure. d, Benzene breakthrough curves for BUT-55 and Co(BDP) recorded at different relative humidities at 298 K. Source data

BUT-53 to BUT-58 samples were examined for their benzene adsorption properties. Single-component benzene adsorption isotherms collected at 298 K for various equilibration times indicated that a long equilibration time of 1,000 s (with the pressure change less than 0.1% of the average pressure during this interval) was required to reach equilibrium at low pressures (Fig. 3a,b and Supplementary Figs. 19–21). The resulting isotherms revealed that benzene uptake by BUT-53 to BUT-56 and BUT-58 sharply increased at low pressure before gradually increasing at higher pressures (Fig. 3a,b). For BUT-57, two sharp steps were observed: one at low pressure, the other at *P*/*P*_0_ = 0.4 (Fig. 3a). The second step suggests structural flexibility triggered by benzene, a feature of a growing number of MOFs, including Co(BDP). At low benzene pressure, the following adsorption uptakes were observed: 2.47 mmol g^−1^ at 5.0 Pa for BUT-53, 2.61 mmol g^−1^ at 9.9 Pa for BUT-54, 3.28 mmol g^−1^ at 7.3 Pa for BUT-55, 2.98 mmol g^−1^ at 6.9 Pa for BUT-56, 3.01 mmol g^−1^ at 9.1 Pa for BUT-57 and 3.09 mmol g^−1^ at 9.5 Pa for BUT-58 (Fig. 3b).

**Fig. 4:**
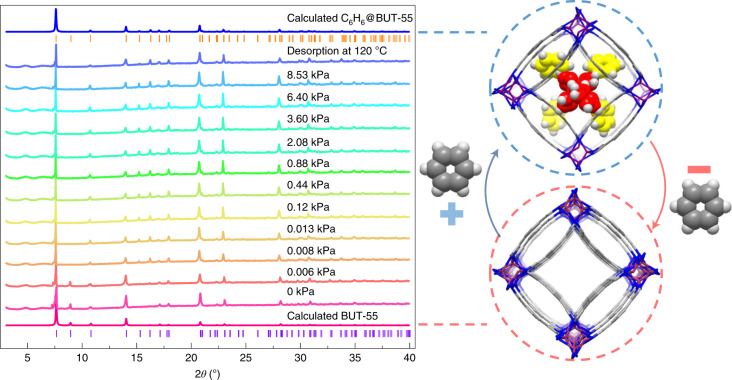
In situ variable-pressure PXRD patterns of BUT-55. PXRD patterns collected under benzene pressure from 0 to 8.53 kPa (guest-free to benzene-loaded BUT-55, with structures shown on the right) and after desorption at 120 °C. The data indicate that no structural transformation occurs during the loading and unloading of benzene. The vertical lines shown below the two calculated PXRD patterns are Bragg positions based on the corresponding single crystal structures. Source data

The steps observed in the isotherms of BUT-55 and BUT-58 in the *P*/*P*_0_ range 0.0001–0.001 could indicate a structural change triggered by benzene loading (Fig. 3b). However, in situ PXRD patterns collected from activated BUT-55 at various benzene pressures and temperatures (Fig. 4 and Supplementary Fig. 22) suggest a rigid structure. Therefore, although steps are seen in the isotherms of flexible MOF structures^42^, we attribute the steps observed here for BUT-55 and BUT-58 to pore filling. BUT-53 to BUT-58 outperform previously studied benzene adsorbents^9,18–25,27,39,43–45^ such as MIL-101(Cr) (0.5 mmol g^−1^ at 4.9 Pa)^18^, HKUST-1 (1.0 mmol g^−1^ at 50.0 Pa)^43^, PAF-1 (3.30 mmol g^−1^ at 75.0 Pa)^21^, BUT-66 (2.83 mmol g^−1^ at 58.0 Pa)^21^ and Carboxen 1000 (2.80 mmol g^−1^ at 53.0 Pa^21^; Fig. 3c). BUT-53 started to adsorb benzene at the lowest pressure (0.53 Pa), but its uptake of 2.47 mmol g^−1^ was relatively low, and although BUT-54 exhibited the highest uptake, 4.36 mmol g^−1^ at 151 Pa, this was achieved at the highest loading pressure. Importantly, BUT-53 to BUT-58 each exhibited strong uptake (>2 mmol g^−1^) of benzene below 10 Pa (Fig. 3b). Overall, the stability and high uptake of BUT-53 to BUT-58 at low pressure make them candidates for the trace removal of benzene from air.

Practically, benzene capture from air will occur in the presence of humidity, which may impact the performance of adsorbents by the co-adsorption of water vapour. Water vapour adsorption experiments at 298 K were performed to assess the hydrophobicity of the MOFs. Supplementary Fig. 23 reveals that each sorbent exhibited S-shaped water adsorption isotherms at >50% relative humidity (RH), except for BUT-56, which exhibited a gradual uptake from 30% RH. That BUT-56 is the most hydrophilic variant is attributed to the pyridyl moiety. For BUT-53 to BUT-55, BUT-57 and BUT-58, a step at *P*/*P*_0_ ≥ 0.6 and hysteresis were observed, suggesting that, except for BUT-56, the BUT family excludes water below 60% RH.

To evaluate the performance of BUT-53 to BUT-58 for capturing trace benzene from ambient air we conducted dynamic gas breakthrough experiments (Supplementary Fig. 24). An air mixture containing trace benzene (10 ppm) was passed through a column packed with ~10 mg of BUT sorbent at a gas flow rate of 10 ml min^−1^. Control experiments were also performed at RH 50 and 80% at 298 K (Supplementary Figs. 25–29). Supplementary Fig. 25 reveals that benzene broke through the BUT-53 column after ~57 h (5,700 h g^−1^) for dry gas, a benzene capture capacity of ~1.53 mmol g^−1^. BUT-53 exhibited reduced capacity (4,600 h g^−1^ and 1.23 mmol g^−1^) at RH 50%, and further reduced capacity (2,200 h g^−1^ and 0.59 mmol g^−1^) at RH 80%. BUT-54 exhibited a breakthrough time of 3,000 h g^−1^ (0.80 mmol g^−1^) under dry conditions and 1,500 h g^−1^ (0.40 mmol g^−1^) at RH 50 and 80% (Supplementary Fig. 26). A breakthrough time of 80 h (8,000 h g^−1^) and a benzene capacity of 2.14 mmol g^−1^ were recorded for BUT-55 under dry conditions and 50% RH (Fig. 3d), with a reduced adsorption capacity (6,000 h g^−1^ and 1.61 mmol g^−1^) at 80% RH. BUT-56 demonstrated the same capacity as BUT-55 under dry conditions, but was more affected by humidity (4,600 h g^−1^ and 1.23 mmol g^−1^ at 50% RH, and 2,700 h g^−1^ and 0.72 mmol g^−1^ at 80% RH; Supplementary Fig. 27). BUT-57 broke through after 40 h (4,000 h g^−1^ and 1.07 mmol g^−1^) under both dry conditions and RH 50%, whereas at 80% RH it occurred at 23 h (2,300 h g^−1^ and 0.62 mmol g^−1^; Supplementary Fig. 28). BUT-58 behaved similarly to BUT-55 in the breakthrough experiments (7,900 h g^−1^ and 2.11 mmol g^−1^ for dry gas, 7,500 h g^−1^ and 2.01 mmol g^−1^ at 50% RH, and 5,000 h g^−1^ and 1.34 mmol g^−1^ at 80% RH; Supplementary Fig. 29).

Compared with the results obtained under dry conditions, decreased breakthrough times at RH 80% were observed for BUT-53, BUT-54, BUT-56 and BUT-57 (Supplementary Figs. 25–28). However, BUT-55 (8,000 h g^−1^ and 2.14 mmol g^−1^ under dry conditions compared with 6,000 h g^−1^ and 1.61 mmol g^−1^ at RH 80%; Fig. 3d) and BUT-58 (7,500 h g^−1^ and 2.01 mmol g^−1^ under dry conditions compared with 5,000 h g^−1^ and 1.34 mmol g^−1^ at RH 80%; Supplementary Fig. 29) were less affected by humidity and were studied for their benzene adsorption performance after exposure to moisture for 1 week. BUT-55 and BUT-58 exhibited similar isotherms to those obtained from pristine samples (Supplementary Fig. 30).

In addition to humidity, polluted air might contain other VOCs with similar physicochemical properties to benzene, such as cyclohexane and ethanol. BUT-55 exhibited cyclohexane and ethanol uptakes of 2.90 mmol g^−1^ at 8.4 Pa and 4.10 mmol g^−1^ at 94 Pa, respectively (Supplementary Figs. 31a,b and 32a,b). The single-component breakthrough curves indicated a cyclohexane adsorption capacity of 1.60 mmol g^−1^ but little ethanol uptake at 10 ppm (Supplementary Figs. 31c and 32c). In competition tests with benzene (1:1, v/v), the adsorption capacity of cyclohexane decreased to 1.28 mmol g^−1^, and ethanol broke through immediately (Supplementary Figs. 31d and 32d). Benzene adsorption capacity decreased slightly in the presence of cyclohexane (1.60 mmol g^−1^) and ethanol (1.60 mmol g^−1^). These results indicate that the tested VOCs have a minor effect upon the benzene adsorption performance of BUT-55.

The residual benzene in effluent streams flowing through a BUT-55 packed bed was determined to be 2.82 ng l^−1^ (2.82 ng l^−1^ = 2.82 μg m^−3^) by gas chromatography mass spectrometry (GC–MS; Supplementary Figs. 33 and 34 and Supplementary Table 2). These data indicate benzene removal to a level below indoor limits (3–100 μg m^−3^) set by most countries/regions, including China, the European Union and the United States^4,46,47^. Recycling breakthrough tests revealed that BUT-55 showed no loss of benzene capture capacity after five cycles (Supplementary Fig. 35), and adsorbed benzene molecules could be removed at 120 °C within 2 h (Supplementary Fig. 36).

The contrast between Co(BDP)/Zn(BDP) and BUT-55/BUT-58 was addressed by preparing Co(BDP)/Zn(BDP) and activating them following the reported approach^39,40^. Their PXRD patterns and N_2_ adsorption isotherms matched those in previous reports (Supplementary Figs. 14 and 15). Single-component benzene vapour adsorption and dynamic breakthrough experiments revealed uptake of 1.8 mmol g^−1^ at 32 Pa (Supplementary Fig. 37) and a benzene capture capacity of 0.17 mmol g^−1^ for Co(BDP) (Fig. 3d), whereas Zn(BDP) exhibited an uptake of 1.3 mmol g^−1^ at 1.0 Pa (Supplementary Fig. 38) and a benzene capture capacity of 0.17 mmol g^−1^ (Supplementary Fig. 29). These values are much lower than those obtained for BUT-55 and BUT-58.

### Benzene binding sites

**Fig. 5:**
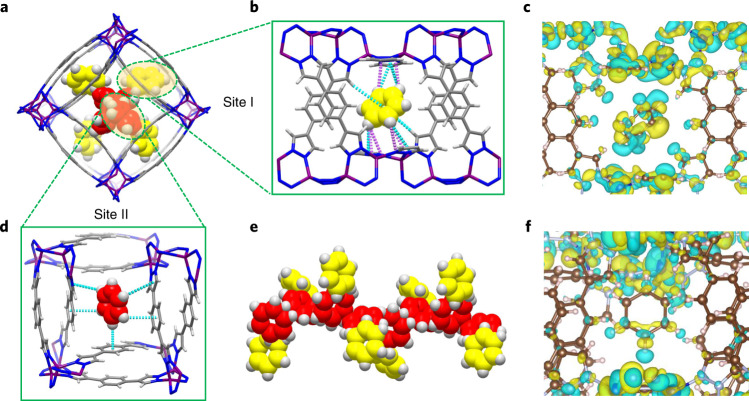
SCXRD structure of C_6_H_6_@BUT-55 and DFT calculations. **a**, Adsorbed benzene molecules lie in pores, as viewed along the *c* axis. **b**,**d**, Binding sites of adsorbed benzene molecules at site I (**b**) and site II (**d**), with C–H···π interactions presented in blue and C–H···N interactions in pink. **e**, Benzene–benzene interactions occur between the two types of adsorbed benzene molecules. **c**,**f**, Isosurfaces of the charge density difference for the interactions of site I (**c**) and site II (**f**) with adsorbed benzene molecules in BUT-55. Yellow indicates increased electronic density and light blue indicates depletion.

To gain insight into the benzene capture performance of the BUT family we determined the single-crystal structures of several guest-loaded phases, including benzene-loaded C_6_H_6_@BUT-53, C_6_H_6_@BUT-54, C_6_H_6_@BUT-55 and C_6_H_6_@BUT-58, water-loaded H_2_O@BUT-55, and benzene- and water-loaded C_6_H_6_/H_2_O@BUT-55. The crystal structure of C_6_H_6_@BUT-55 reveals that the framework was little changed after the capture of benzene, and two types of adsorbed benzene molecules were identified (Fig. 5). One (yellow) resides in the cavity between two neighbouring pairs of BDP^2−^ ligands and two Co(pz)_2_ RBBs (site I; Fig. 5a,b). This benzene molecule binds to surrounding BDP^2−^ ligands through multiple C–H···X interactions (distances 2.89–3.58 Å): to pyrazolate C–H moieties through C–H(L)···π(B) interactions (L represents BDP^2−^ ligands and B represents benzene guest molecules), to pyrazolate π-electrons through C–H(B)···π(L) interactions and to pyrazolate nitrogen atoms through C–H(B)···N(L) hydrogen bonds (Fig. 5b and Supplementary Table 3)^48^. The other benzene molecule (red) lies over a pair of packed BDP^2−^ ligands (site II; Fig. 5a,d), and also exhibits multiple C–H···π interactions with adjacent BDP^2−^ ligands (distances 2.78–3.88 Å; Fig. 5d and Supplementary Table 3). In the square unit defined by four rectangular pores and four Co(pz)_2_ RBBs, the criss-cross packed BDP^2−^ ligand pairs are arranged along the channel walls in a helical mode. Consequently, these (red) benzene molecules form a helical chain. Guest–guest binding occurs through C–H(B)···π(B) interactions (Fig. 5e). Collectively, this plethora of host–guest and guest–guest C–H···X interactions drive the adsorption of benzene by the host framework. The single-crystal structure of C_6_H_6_@BUT-55 suggests that benzene uptake in BUT-55 should be 3.43 mmol g^−1^, consistent with the uptake observed in the adsorption isotherms (3.62 mmol g^−1^ at P/P0 = 1; Fig. 3a,b).

To further understand the observed adsorption behaviour, DFT calculations were conducted on the basis of the experimental single-crystal structures. Isosurfaces of charge density differences before and after benzene loading in BUT-55 are shown in Fig. 5c,f and Supplementary Fig. 39. Figure 5c,f reveals that benzene molecules interact more strongly in site I through electron accumulation on benzene and depletion on the ligand. Preferential benzene adsorption at site I is supported by a higher calculated binding energy of −110.06 kJ mol^−1^ (−72.99 kJ mol^−1^ at site II). At lower pressures of benzene, benzene molecules may first bind to BUT-55 at site I. Similar results were obtained for BUT-53, BUT-54 and BUT-58 (Supplementary Table 3 and Supplementary Figs. 40–42). That BUT-53 was calculated to exhibit the strongest benzene binding at site I agrees with its experimental adsorption profile at ultralow pressure (that is, the first few data points in Fig. 3b and Supplementary Fig. 43a). However, the isotherm for BUT-55 crosses that of BUT-53 at low pressure and offers better benzene sorption performance than BUT-53 at higher loading (Supplementary Fig. 43a and Supplementary Section 7). The performance of BUT-55 could be attributed to slightly faster kinetics of loading compared with BUT-53 (Supplementary Fig. 43b), but we also note that benzene binding induced a minor structural change, ligand swing, in BUT-53 (Supplementary Fig. 43c).

The impact of moisture on the benzene adsorption ability of BUT-55 was addressed by analysis of both the single-crystal structural data of H_2_O@BUT-55 and C_6_H_6_/H_2_O@BUT-55 and calculated data (Supplementary Fig. 44). In the single-crystal structure of H_2_O@BUT-55, water molecules are located at site I (Supplementary Fig. 44a,b), but no water molecules are observed at site II because of unresolvable crystallographic disorder. In the structure of C_6_H_6_/H_2_O@BUT-55, water molecules are located at site I and benzene molecules are found at site II (Supplementary Fig. 44d). We also calculated the energy of interaction between BUT-55 and water molecules. DFT calculations indicate the binding energy for water molecules to be −31.51 to −43.52 kJ mol^−1^, much lower than for benzene (−110.06 kJ mol^−1^ at site I and −72.99 kJ mol^−1^ at site II). Preferential benzene binding in the co-adsorption of benzene and moisture would therefore be expected in breakthrough experiments performed under humid conditions. These results provide insight as to why moisture has a relatively small effect on the benzene adsorption performance of BUT-55 (Fig. 3d).

In conclusion, a family of double-walled pyrazolate MOFs, BUT-53 to BUT-58, capture trace benzene from air at 298 K owing to benzene-selective binding sites resulting from a plethora of weak non-covalent interactions, including C–H···X interactions. BUT-55 is notable because it performs well even under humid conditions, which we attribute to its relatively low affinity for water molecules. BUT-55 can therefore reduce trace benzene concentrations in air to levels below acceptable limits and, as a physisorbent, requires relatively low energy for recycling. This work further highlights that the tuning of pore chemistry and pore size can afford tight binding sites that enable physisorbents to remove even trace contaminants from air with advantageously long breakthrough times.

## Methods

### Materials and instruments

All reagents and solvents (AR grade) were purchased commercially and used as received. ^1^H NMR spectra were recorded on a BRUKER AVANCE III HD 400 MHz spectrometer. PXRD patterns were recorded on a Rigaku Smartlab 3 X-ray powder diffractometer using a Cu sealed tube (*λ* = 1.54178 Å) at room temperature. In situ variable-pressure PXRD patterns were collected on a Rigaku Smart Lab instrument at 40 kV and 40 mA using an Anton Paar TTK 600 accessory. TGA data were obtained on a SHIMADZU TGA-50 thermogravimetric analyser at a heating rate of 10 °C min^–1^ under air. Gas adsorption–desorption isotherms were obtained using a Micrometrics ASAP 2020 or BEL BELSORP-maxII instrument. Column breakthrough experiments were carried out using a typical breakthrough system consisting of a gas source, adsorption bed and gas analyser. The residual benzene in effluent was determined using a Clarus 600 GC–MS instrument (Perkin Elmer) coupled with a Turbomatrix350 thermal detector (Perkin Elmer).

### Synthesis of ligands

The synthetic procedures for the ligands H_2_DPB, H_2_DPN, H_2_BDP, H_2_DPP and H_2_PDP are described in detail in Supplementary Section 1 and Supplementary Figs. 45–54).

### Synthesis of BUT-53 (CoDPB)

H_2_DPB (0.14 mmol, 30 mg) and Co(OAc)_2_·4H_2_O (0.24 mmol, 60 mg) were dissolved under ultrasound in 10 ml DMF in a 20-ml glass vial. Next, 0.10 ml acetic acid and 8.0 ml deionized water were added to the mixture. The vial was then tightly sealed and the mixture sonicated for another 15 min. The resulting suspension was heated in a traditional oven at 150 °C for 12 h. After cooling naturally to room temperature, bluish violet crystals were filtered off and washed with DMF (2 × 15 ml) and acetone (9 × 15 ml) with short cycles of ultrasonic treatment to remove amorphous solids. The crystals (15 mg of activated sample, 49% yield based on the H_2_DPB ligand) were collected by filtration and dried under vacuum at 100 °C for 5 h.

### Synthesis of BUT-54 (CoDPN)

H_2_DPN (0.090 mmol, 24 mg) and Co(OAc)_2_·4H_2_O (0.16 mmol, 40 mg) were dissolved under ultrasound in 8.0 ml DMF in a 20-ml glass vial. Next, 0.080 ml acetic acid and 2.4 ml deionized water were added to the mixture. The vial was then tightly sealed and the mixture sonicated for another 15 min. The resulting suspension was heated in a traditional oven at 150 °C for 24 h. After cooling naturally to room temperature, wine-red crystals were filtered off and washed with DMF (2 × 15 ml) and acetone (9 × 15 ml) with short cycles of ultrasonic treatment to remove amorphous solids. The crystals (19 mg of activated sample, 65% yield based on the H_2_DPN ligand) were collected by filtration and dried under vacuum at 100 °C for 5 h.

### Synthesis of BUT-55 (CoBDP)

H_2_BDP (0.11 mmol, 24 mg) and Co(OAc)_2_·4H_2_O (0.19 mmol, 48 mg) were dissolved under ultrasound in 8.0 ml DMF in a 20-ml glass vial. Next, 4.0 ml deionized water was added to the mixture. The vial was then tightly sealed and the mixture sonicated for another 15 min. The resulting suspension was heated in a traditional oven at 150 °C for 12 h. After cooling naturally to room temperature, wine-red crystals were filtered off and washed with DMF (2 × 15 ml) and acetone (9 × 15 ml) with short cycles of ultrasonic treatment to remove amorphous solids. The crystals (23 mg of activated sample, 75% yield based on the H_2_BDP ligand) were collected by filtration and dried under vacuum at 100 °C for 5 h.

### Synthesis of BUT-56 (CoDPP)

H_2_DPP (0.11 mmol, 24 mg) and Co(OAc)_2_·4H_2_O (0.19 mmol, 48 mg) were dissolved under ultrasound in 8.0 ml DMF in a 20-ml glass vial. Next, 4.0 ml deionized water was added to the mixture. The vial was then tightly sealed and the mixture sonicated for another 15 min. The resulting suspension was heated in a traditional oven at 150 °C for 12 h. After cooling naturally to room temperature, wine-red crystals were filtered off and washed with DMF (2 × 15 ml) and acetone (9 × 15 ml) with short cycles of ultrasonic treatment to remove amorphous solids. The crystals (22 mg of activated sample, 72% yield based on the H_2_DPP ligand) were collected by filtration and dried under vacuum at 100 °C for 5 h.

### Synthesis of BUT-57 (CoPDP)

H_2_PDP (0.090 mmol, 30 mg) and Co(OAc)_2_·4H_2_O (0.24 mmol, 60 mg) were dissolved under ultrasound in 10 ml DMF in a 20-ml glass vial. Next, 0.050 ml acetic acid and 1.5 ml deionized water were added to the mixture. The vial was then tightly sealed and the mixture sonicated for another 15 min. The resulting suspension was heated in a traditional oven at 120 °C for 12 h. After cooling naturally to room temperature, wine-red crystals were filtered off and washed with DMF (2 × 15 ml) and acetone (9 × 15 ml) with short cycles of ultrasonic treatment to remove amorphous solids. The crystals (21 mg of activated sample, 60% yield based on the H_2_PDP ligand) were collected by filtration and dried under vacuum at 100 °C for 5 h.

### Synthesis of BUT-58 (ZnBDP)

H_2_BDP (0.090 mmol, 30 mg) and Zn(NO_3_)_2_·6H_2_O (0.24 mmol, 60 mg) were dissolved under ultrasound in 10 ml DMF in a 20-ml glass vial. Next, 7.0 ml deionized water was added to the mixture. The vial was then tightly sealed and the mixture sonicated for another 15 min. The resulting suspension was heated in a traditional oven at 100 °C for 6 h. After cooling naturally to room temperature, colourless crystals were filtered off and washed with DMF (2 × 15 ml) and acetone (9 × 15 ml) with short cycles of ultrasonic treatment to remove amorphous solids. The crystals (17 mg of activated sample, 44% yield based on the H_2_BDP ligand) were collected by filtration and dried under vacuum at 100 °C for 5 h.

### Synthesis of Co(BDP)

H_2_BDP (0.14 mmol, 30 mg) and Co(NO_3_)_2_·4H_2_O (0.23 mmol, 60 mg) were dissolved under ultrasound in 10 ml DMF in a 20-ml glass vial. Next, 2.0 ml deionized water was added to the solution. The vial was then tightly sealed and the mixture sonicated for another 15 min. The resulting solution was heated in a traditional oven at 130 °C for 12 h. After cooling naturally to room temperature, blue crystals were filtered off and washed with DMF (2 × 15 ml) and acetone (9 × 15 ml) with short cycles of ultrasonic treatment to remove amorphous solids. The crystals (18 mg of activated sample, 47% yield based on the H_2_BDP ligand) were collected by filtration and dried under vacuum at 100 °C for 5 h.

### Synthesis of Zn(BDP)

H_2_BDP (0.14 mmol, 30 mg) and Zn(NO_3_)_2_·6H_2_O (0.23 mmol, 60 mg) were dissolved under ultrasound in 10 ml DMF in a 20-ml glass vial. Next, 2.0 ml deionized water was added to the solution. The vial was then tightly sealed and the mixture sonicated for another 15 min. The resulting solution was heated in a traditional oven at 80 °C for 6 h. After cooling naturally to room temperature, colourless crystals were filtered off and washed with DMF (2 × 15 ml) and acetone (9 × 15 ml) with short cycles of ultrasonic treatment to remove amorphous solids. The crystals (18 mg of activated sample, 46% yield based on the H_2_BDP ligand) were collected by filtration and dried under vacuum at 100 °C for 5 h.

### Single-crystal X-ray diffraction

The diffraction data of as-synthesized BUT-53 to BUT-58 samples and guest-loaded phases were collected on a Rigaku Supernova CCD diffractometer (mirror monochromator, Cu Kα source, *λ* = 1.54184 Å).

To obtain single crystals of the benzene-loaded phases, open vials each containing an activated BUT-53 to BUT-58 sample were loaded into a bottle containing an open vial of benzene and the bottle was then sealed. H_2_O@BUT-55 was obtained by loading activated BUT-55 in an open vial into a bottle containing an open vial of water and the bottle was sealed. For the C_6_H_6_/H_2_O@BUT-55 phase, an open vial containing activated BUT-55 was loaded into a bottle containing open vials of both water and benzene, and the bottle was then sealed. After 24 h, single crystals of C_6_H_6_@BUT-53, C_6_H_6_@BUT-54, C_6_H_6_@BUT-55, C_6_H_6_@BUT-58, H_2_O@BUT-55 and C_6_H_6_/H_2_O@BUT-55 were harvested and studied by SCXRD. Both H_2_O@BUT-55 and C_6_H_6_/H_2_O@BUT-55 were obtained under 100% RH. The specific binding sites of benzene could hardly be determined in the single-crystal structure of C_6_H_6_@BUT-56, and single crystals of C_6_H_6_@BUT-57 were of insufficient quality to be characterized by SCXRD.

### Sample activation and gas adsorption

Sample activation and gas adsorption measurements were performed according to ref. ^49^ and the details are reproduced here for completeness. Prior to the gas adsorption tests, samples of BUT-53 to BUT-58 (~80 mg of each) were immersed in 15 ml DMF at 60 °C for 24 h; the solvent was exchanged for fresh DMF after 12 h. The samples were then harvested carefully by decanting and next soaked in 15 ml acetone at room temperature for another 72 h, during which time the solvent was exchanged for fresh acetone three times a day. After this time, the samples were loaded into a sample tube and degassed under high vacuum at an optimal temperature of 100 °C for 5 h.

N_2_ and water vapour adsorption isotherms were collected on a Micromeritics ASAP 2020 surface area and pore analyser, which was customized with all stainless-steel parts inside the manifold. Benzene vapour adsorption isotherms were recorded on a Micromeritics ASAP 2020 or BELSORP-maxII vapour adsorption instrument. The adsorption temperature was controlled using a liquid nitrogen bath (77 K) or a water bath (298 K).

### Breakthrough experiments

Breakthrough experiments were performed according to ref. ^21^ and the details are reproduced here for completeness. A calibration gas (~20 ppm benzene balanced with dry air) and a set-up as shown in Supplementary Fig. 24 were used. Quartz tubes (3.0 mm outer diameter, 1 mm inner diameter and 50 mm length) were packed with the samples. For dry gas breakthrough experiments, a mixture of the calibration gas and dry air (1:1, v/v) was passed through the packed tube at a flow rate of 10 ml min^−1^. The 50% RH breakthrough experiments were performed similarly to the dry gas experiments except that the dry air flow was presaturated with water vapour by bubbling through deionized water before being mixed with the calibration gas flow. For the 80% RH experiments, a calibration gas containing 50 ppm benzene was used, and the ratio of calibration gas to humid air was set to 1:4 (v/v) with a total flow rate of 10 ml min^−1^. The breakthrough experiments were all conducted with a feed stream containing 10 ppm benzene. The flow rate of the gases was controlled by a mass flow controller (Alicat). The concentration of benzene in the gases passing through the packed columns was monitored using a Hiden HPR20 mass spectrometer gas analysis system.

## Online content

Any methods, additional references, Nature Research reporting summaries, source data, extended data, supplementary information, acknowledgements, peer review information; details of author contributions and competing interests; and statements of data and code availability are available at 10.1038/s41563-022-01237-x.

## Supplementary information

Supplementary Information Supplementary methods, general characterizations and additional tables and figures.

## Data Availability

Crystallographic data for the structures reported in this article have been deposited at the Cambridge Crystallographic Data Centre, under deposition numbers CCDC 2085589 (BUT-53), 2085590 (BUT-54), 2085591 (BUT-55), 2085592 (BUT-56), 2085593 (BUT-57), 2109769 (BUT-58), 2085594 (C_6_H_6_@BUT-55), 2122629 (C_6_H_6_@BUT-54), 2122630 (C_6_H_6_@BUT-53), 2122631 (C_6_H_6_@BUT-58), 2122645 (H_2_O@BUT-55) and 2122646 (C_6_H_6_/H_2_O@BUT-55). Copies of the data can be obtained free of charge via https://www.ccdc.cam.ac.uk/structures/. All other data supporting the findings of this study are available within the article and its Supplementary Information. Source data are provided with this paper.

## Source data

Source Data Fig. 3 Source data for Fig. 3.
Source Data Fig. 4 Source data for Fig. 4.
