# Supplementary material for: Trace removal of benzene vapour using double-walled metal–dipyrazolate frameworks
Source: Nat Mater. 2022 Apr 28;21(6):689–95. doi: 10.1038/s41563-022-01237-x (PMC9156410; doi:10.1038/s41563-022-01237-x)
Supplement: Supplementary file 1 — Supplementary methods, general characterizations and additional tables and figures. [file 41563_2022_1237_MOESM1_ESM.pdf]

nature portfolio

https://doi.org/10.1038/s41563-022-01237-x

Supplementary information

Trace removal of benzene vapour using double-walled metal–dipyrazolate frameworks

In the format provided by the authors and unedited

Supplementary Information

Trace removal of benzene vapor using double-walled metal-dipyrazolate frameworks

Tao He[1,2,6,7], Xiang-Jing Kong[1,3,7], Zhen-Xing Bian[1], Yong-Zheng Zhang[1], Guang-Rui Si[1], Lin-Hua Xie[1], Xue-Qian Wu[1], Hongliang Huang[4], Ze Chang[5], Xian-He Bu[5], Michael J. Zaworotko[3]*, Zuo-Ren Nie[2]*, and Jian-Rong Li[1,2]*

[1] Beijing Key Laboratory for Green Catalysis and Separation and Department of Environmental Chemical Engineering, Beijing University of Technology, Beijing 100124, China

[2] The Key Laboratory of Advanced Functional Materials, Ministry of Education, Faculty of Materials and Manufacturing, Beijing University of Technology, Beijing 100124, China

[3] Bernal Institute and Department of Chemical Sciences, University of Limerick, Limerick, V94 T9PX, Ireland

[4] State Key Laboratory of Separation Membranes and Membrane Processes, Tianjin Polytechnic University, Tianjin 300387, China

[5] School of Materials Science and Engineering and TKL of Metal and Molecule-Based Material Chemistry, Nankai University, Tianjin 300350, China

[6] College of Chemistry and Chemical Engineering, Qingdao University, Shandong 266071, China

[7] These authors contributed equally to this work

Email: xtal@ul.ie; zrnie@bjut.edu.cn; jrli@bjut.edu.cn

Contents

Section 1. Ligand Synthesis.. S3

Section 2. Comparison of synthetic conditions S6

Section 3. X-ray Crystallographic Analysis... S7

Section 4. Gas Adsorption S9

Section 5. *In situ* powder X-ray diffraction....................................... S10

Section 6. Breakthrough Experiments S11

Section 7. First-Principles Based Computational Details S13

Section 8. Additional Figures and Tables... S15

Section 9. References S48

Section 1. Ligand Synthesis

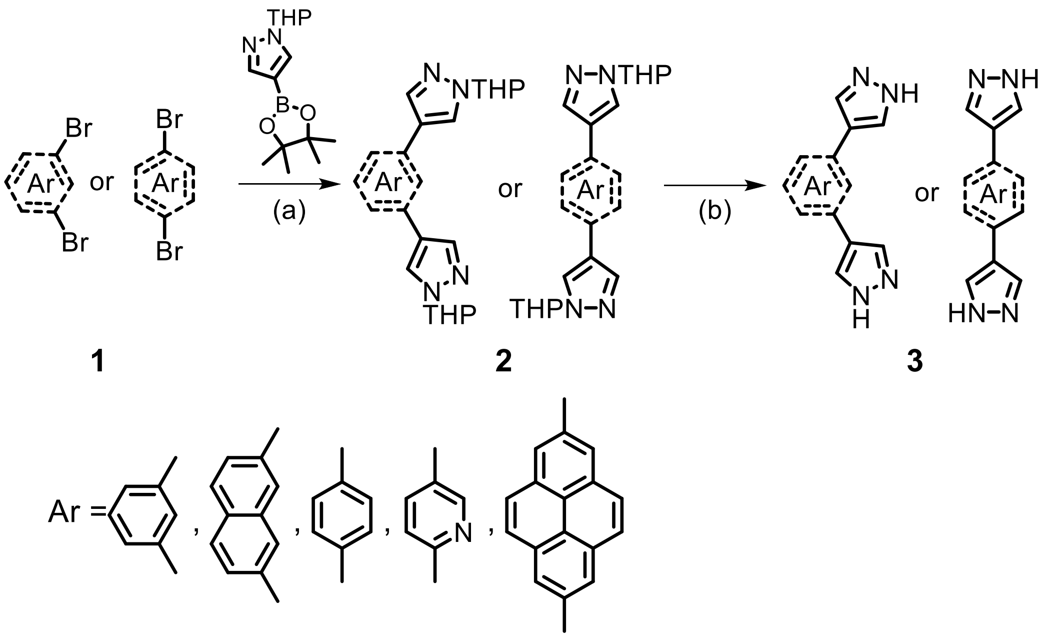

Scheme S1. Synthesis of di-topic pyrazole ligands: (a)K_2CO_3, 1,4-dioxane, H_2O, $Pd(PPh_3)_4$; (b) EtOH, HCl.

General Suzuki coupling procedure.[S1] To the mixture of **1** (10 mmol) and 1-(tetrahydro-2H-pyran-2-yl)-4-(4,4,5,5-tetramethyl-1,3,2-dioxaborolan-2-yl)-1H-pyrazole (40 mmol) in 1,4-dioxane (240 mL) and water (60 mL) was added K_2CO_3 (40 mmol) and $Pd(PPh_3)_4$ (2 mmol) under nitrogen atmosphere. The reaction mixture was stirred at 110 ^oC for 48 h. After the starting materials were consumed, the solvent was removed and the residue was dissolved in ethyl acetate (300 mL), washed with water (300 mL × 2) and brine (300 mL), dried over anhydrous Na_2SO_4, filtered and concentrated. The crude product was purified by column chromatography to give **2**.

General deprotection procedure.[S1] The solution of **2** (5 mmol) in ethanol (200 mL) and concentrated HCl (5 mL) was stirred at 50 ^oC for 12 h. The organic solvent was removed by rotary evaporation. The solid was dissolved in water (100 mL), and the pH was adjusted to around 10 by progressively adding saturated solution of Na_2CO_3. The mixture was filtered and washed with water (100 mL × 3). The solid was dried under vacuum at 60 ^oC to give **3**.

2.1 Synthesis of 1,3-di(1*H*-pyrazol-4-yl)benzene (H_2DPB)

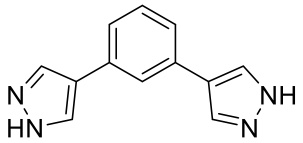

DPB-THP: ^{1}H NMR (400 MHz, $CDCl_3$) δ 7.91 (2H, s), 7.86 (2H, s), 7.61 (1H, s), 7.36 (3H, s), 5.42-5.45 (2H, m), 4.09-4.13 (2H, m), 3.71-3.77 (2H, m), 2.05-2.14 (6H, m), 1.64-1.75 (6H, m) (Figure S45).

H_2DPB: ^{1}H NMR (100 MHz, DMSO-d_6) δ 8.15 (4H, s), 7.86-7.87 (1H, m), 7.41-7.44 (2H, m), 7.30-7.34 (1H, m) (Figure S46).

2.2 Synthesis of 2,7-di(1H-pyrazol-4-yl)naphthalene (H_2DPN)

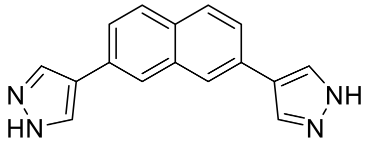

DPN-THP: ^{1}H NMR (400 MHz, $CDCl_3$) δ 8.00 (2H, s), 7.98 (2H, s), 7.93 (2H, s), 7.80-7.82 (2H, m), 7.59 (2H, dd, *J* = 1.6, 8.4 Hz), 5.46 (2H, dd, *J* = 3.0, 9.2 Hz), 4.11-4.14 (2H, m), 3.73-3.79 (2H, m), 2.17-2.20 (6H, m), 1.66-1.77 (6H, m) (Figure S47).

H_2DPN: ^{1}H NMR (100 MHz, DMSO-d_6) δ 12.97 (2H, s), 8.19 (4H, s), 8.06 (2H, s), 7.84-7.86 (2H, m), 7.71-7.74 (2H, m) (Figure S48).

2.3 Synthesis of 1,4-di(1H-pyrazol-4-yl)benzene (H_2BDP)

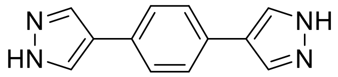

BDP-THP: ^{1}H NMR (400 MHz, $CDCl_3$) δ 7.88 (2H, s), 7.84-7.85 (2H, m), 7.50 (4H, s), 5.43 (2H, dd, *J* = 3.6, 8.8 Hz), 4.11 (2H, d, *J* = 2.0, 8.0 Hz), 3.71-3.78 (2H, m), 2.06-2.17 (6H, m), 1.65-1.76 (6H, m) (Figure S49).

H_2BDP: ^{1}H NMR (100 MHz, DMSO-d_6) δ 12.78 (2H, s), 8.06 (4H, s), 7.58 (4H, s) (Figure S50).

2.4 Synthesis of 2,5-di(1H-pyrazol-4-yl)pyridine (H_2DPP)

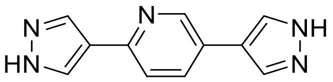

DPP-THP: ^{1}H NMR (400 MHz, $CDCl_3$) δ 8.73 (1H, dd, J = 0.72, 2.4 Hz), 8.19 (1H, s), 8.05 (1H, d, J = 0.48 Hz), 7.93 (1H, s), 7.86 (1H, d, J = 0.56 Hz), 7.76 (1H, dd, J = 2.32, 8.2 Hz), 7.49 (1H, dd, J = 0.68, 8.2 Hz), 5.44-5.47 (2H, m), 4.08-4.14 (2H, m), 3.73-3.79 (2H, m), 2.15-2.17 (6H, m), 1.66-1.76 (6H, m) (Figure S51).

H_2DPP: ^{1}H NMR (100 MHz, DMSO-d_6) δ 13.04 (2H, s), 8.80 (1H, d, J = 1.84 Hz), 8.30 (2H, s), 8.06 (2H, s), 7.96 (1H, dd, J = 2.28, 8.2 Hz), 7.67 (1H, d, J = 8.2 Hz) (Figure S52).

2.5 Synthesis of 2,7-di(1H-pyrazol-4-yl)pyrene (H_2PDP)

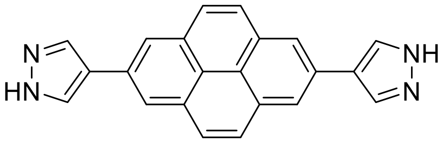

PDP-THP: ^{1}H NMR (400 MHz, $CDCl_3$) δ 8.29 (4H, s), 8.17 (2H, s), 8.13 (2H, s), 8.07 (2H, s), 5.52 (2H, dd, J = 3.08, 9.2 Hz), 4.16 (2H, m), 3.77-3.84 (2H, m), 2.12-2.26 (6H, m), 1.70-1.81 (6H, m) (Figure S53).

H_2PDP: ^{1}H NMR (100 MHz, DMSO-d_6) δ 13.11 (2H, s), 8.52 (4H, s), 8.36 (4H, s), 8.14 (4H, s) (Figure S54).

Section 2. Comparison of synthetic conditions

Based on the H_2BDP ligand, four supramolecular isomers, including the single-walled Co(BDP) and Zn(BDP), and double-walled isomers BUT-55 and BUT-58, have synthesized, respectively. The synthetic conditions are summarized in Table S4 for comparison. It was found that less water favors Co(BDP) and Zn(BDP), whereas more water and higher temperature favors BUT-55 and BUT-58.

Section 3. X-ray Crystallographic Analysis

The datasets were corrected by empirical absorption correction using spherical harmonics, implemented in the SCALE3 ABSPACK scaling algorithm.[S2] All the structures were solved using direct methods and refined by full-matrix least-squares on F^2 with anisotropic displacement using the SHELXTL software package.[S3] Non-hydrogen atoms on the frameworks were refined with anisotropic displacement parameters during the final cycles. The hydrogen atoms on the ligands were positioned geometrically and refined with a riding model. The electron density of the disordered guest molecules was flattened according to the SQUEEZE routine in PLATON.[S4] Crystal parameters and structure refinement of BUT-53 to BUT-58 are summarized in Table S1 (for details, see CCDC 2085589-2085594, 2118113).

Single-crystal structure analysis on H_2O@BUT-55: The single-crystal structure of H_2O@BUT-55 suggests that there should be 1.5 water molecules absorbed per formula at site I, corresponding to 38% of water uptake (5.5 mmol) as reflected by the water sorption isotherm (maximum uptake of 14.4 mmol). Site II must therefore account for the remaining 8.9 mmol of uptake even though no absorbed water molecules were observed in the SCXRD structure H_2O@BUT-55. We recollected the SCXRD data at 100% RH, but obtained similar results (high crystallographic disorder of water molecules at site II). Simulated distribution of adsorbed water molecules in the unit cell of BUT-55 also supports the presence of water molecules at site II (Fig. S44). In summary, water sorption uptake and simulations support the presence of water molecules at both sites I and II.

Table S1. Crystallographic data and structure refinements for BUT-53 to BUT-58.

Name	BUT-53	BUT-54	BUT-55	BUT-56	BUT-57	BUT-58
Formula	$C_{12}H_8N_4Co$	$C_{16}H_{10}N_4Co$	$C_{12}H_8N_4Co$	$C_{11}H_7N_5Co$	$C_{22}H_{12}N_4Co$	$C_{12}H_8N_4Zn$
M	267.15	317.21	267.15	268.00	391.04	273.59
Crystal system	Tetragonal	Tetragonal	Tetragonal	Tetragonal	monoclinic	Tetragonal
Space group	$I4_1/amd$	$I4_1/amd$	$I4_122$	$I4_122$	$I2$	$I4_122$
a/ Å	22.9244(4)	27.6797(5)	16.3606(5)	16.2017(14)	18.1194(7)	16.0793(12)
b/ Å	22.9244(4)	27.6797(5)	16.3606(5)	16.2017(14)	26.4519(8)	16.0793(12)
c/ Å	12.4457(3)	12.2240(2)	12.4068(6)	12.3386(11)	39.0573(7)	12.3447(9)
α/ º	90	90	90	90	90	90
β/ º	90	90	90	90	90.019(3)	90
γ/ º	90	90	90	90	90	90
V/ $Å^3$	6540.6(3)	9365.6(4)	3320.9(3)	3238.8(6)	18719.9(10)	3191.6(5)
Z	16	16	8	8	4	8
D_C /g cm^{-3}	1.085	0.900	1.069	1.096	0.833	1.139
μ /mm^{-1}	8.116	5.729	1.019	8.195	4.369	1.524
T/ K	292.73(10)	291.88(10)	291.63(10)	289.3(2)	293(2)	290.78(10)
Reflections collected	5948	9993	5936	3240	53876	7521
Independent reflections	1705 R_{int} = 0.0340	2415 R_{int} = 0.0549	2050 R_{int} = 0.0538	1531 R_{int} = 0.0467	31670 R_{int} = 0.0857	1990 R_{int} = 0.1191
Goodness-of-fit on F^2	1.032	1.067	0.966	1.112	1.083	1.026
R_1[a], wR_2[b] [$I > 2\sigma(I)$]	R_1 = 0.0787 wR_2 = 0.1989	R_1 = 0.0861 wR_2 = 0.2461	R_1 = 0.0415 wR_2 = 0.0629	R_1 = 0.0779 wR_2 = 0.1972	R_1 = 0.1165 wR_2 = 0.2984	R_1 = 0.0701 wR_2 = 0.1697
R_1[a], wR_2[b] (all data)	R_1 = 0.0893 wR_2 = 0.2054	R_1 = 0.0992 wR_2 = 0.2582	R_1 = 0.0638 wR_2 = 0.0695	R_1 = 0.0985 wR_2 = 0.2155	R_1 = 0.1773 wR_2 = 0.3833	R_1 = 0.1070 wR_2 = 0.2024
Largest diff. peak and hole (e.$Å^{-3}$)	0.71 / –0.69	0.44 / –0.43	0.29 / –0.21	0.87/–0.85	1.37/–0.65	1.00/-0.61

[a] $R_1=\Sigma||F_o|-|F_c|| / \Sigma|F_o|$

[b] $wR_2=\{\Sigma[w(F_o^2-F_c^2)^2]/[w(F_o^2)^2]\}^{1/2}$, $[F_o>4\sigma(F_o)]$

Section 4. Gas Adsorption

Benzene adsorption isotherms shown in Fig. S19 were collected on a Micrometrics ASAP 2020 instrument using default equilibrium parameters (pressure change over 100 s of less than 0.01% of the average pressure during the interval). Data collection for each sample require more than three days.

The equilibrium time of benzene adsorption over BUT-55 was optimized on a BELSORP-maxII vapor adsorption instrument, by varying the setting from 300 s/0.3%, 300 s/0.1%, 600 s/0.1% to 1000 s/0.1% (Fig. S20, with the limitation pressure change per equilibration time interval of 0.1%). Benzene adsorption isotherms for all materials have been collected under the condition of 1000 s/0.1% (Fig. 3a and 3b). Cyclohexane and ethanol adsorption isotherms for BUT-55 were collected under the condition of 300 s/0.1% (Figs S31a-b, and S32a-b).

Section 5. *In situ* powder X-ray diffraction

In situ variable pressure PXRD data were recorded on a Rigaku Smart Lab instrument operated at 40 kV and 40 mA with an Anton Paar TTK 600 accessory. The PXRD patterns were collected under benzene loading from 0 to 8.53 kPa at 32 °C. The sample was activated at 120 °C under vacuum condition before testing.

In situ variable temperature PXRD data were recorded on a PANalytical X'Pert Pro-MPD diffractometer equipped with a PIXcel3D detector operated at 40 kV and 40 mA. Anton Paar TTK 450 stage coupled with the Anton Paar TCU 110 Temperature Control Unit was used to record the variable temperature diffractograms. The PXRD patterns were collected under nitrogen atmosphere and variable temperatures from 25 °C to 300 °C. The sample was collected from DMF solution and dried for one day in air.

Section 6. Breakthrough Experiments

After the content of outlet gas reached equilibrium, the adsorption bed was regenerated by N_2 flow (20 mL/min) for 2 hours at 120 ^{o}C.

To evaluate the behavior of materials under real conditions, BUT-55 and BUT-58 were selected to check their benzene adsorption performance after being previously exposed to moisture. The adsorption experiments were performed by exposing BUT-55 and BUT-58 samples to the atmosphere one week before testing (RH ranging from 35 to 64%, at 9 am from 3rd to 10th Oct in Beijing).

The concentration of benzene in effluent has been analysed with a GC-MS instrument (Clarus 600 GC-MS (Perkin Elmer, U.S) instrument coupled with a Turbomtrix350 TD (Perkin Elmer, U.S)). Samples are collected into a Tenax TA sorbent tube for detection, and the mass of analytes could be calculated by referring to the standard curve with a peak area value. Specifically, LOD of this method can be determined by testing seven samples with a similar analyte mass (ng), identifying the specific benzene mass of each sample by referring to the standard curve, and calculating the standard deviation of these mass values. LOD is finally obtained by multiplying the standard deviation with a coefficient 3.14. For benzene in the present research, the standard deviation was calculated to be 1.599 ng by taking seven samples with benzene mass of around 100 ng (Fig. S32 and Table S2), and the LOD was finally determined to be 5.02 ng. As indicated by this LOD value, if we prepare a sample containing benzene more than 5 ng, the detection would be reliable.

Because the concentration of benzene in effluent streams is very low, we tried to continuously collect effluent for a long period to prepare large volume samples. Three effluent samples were prepared by collecting for 24, 48, and 72 hours, with benzene mass of 37, 78, and 138 ng, and concentration of 2.57, 2.71 and 3.19 ng L^{-1}, respectively. The provided concentration of benzene (2.82 ng L^{-1}) in the manuscript has been determined by averaging these values. Both large volume sampling and results averaging increase the precision of detection.

It should be noted that there are two LOD values provided in this research, one is for breakthrough experiments by using a MS detector, and the other is for determining the benzene concentration in effluent with a GC detector. As calculated from the calibrated curves, LOD of breakthrough experiments is about 33 ppb, and that of GC-MS for effluent detection is about 5 ng.

Section 7. First-Principles Based Computational Details

DFT calculations were carried out to explore the active sites and reaction mechanisms using the CP2K code.[S5] All calculations employed a mixed Gaussian and plane-wave basis sets. For the core electrons, norm-conserving Goedecker-Teter-Hutter pseudopotentials[S6-S9] were adopted. The valence electron wave function was expanded in a double-ζ basis set with polarization functions[S10] along with an auxiliary plane wave basis set. Perdew, Burke, and Enzerhof (PBE) exchange-correlation functional[S11] within the generalized gradient approximation was applied. The cutoff energy was set to 400 Ry. The Γ-point only sampling scheme was used in all calculations. The reaction state configurations were optimized with the Broyden-Fletcher-Goldfarb-Shanno (BGFS) algorithm with SCF convergence criteria of 1.0×10^{-8} au. To compensate the long-range van der Waals dispersion interaction between the adsorbates and MOF skeleton, the DFT-D3 scheme[S12] with an empirical damped potential term was added into the energies obtained from the exchange-correlation functional in all calculations.

The adsorption energy between benzene and the BUT-55 substrate can be calculated using the following equation:

$$\Delta E_{ads} = E_{benzene@BUT-55} - E_{benzene} - E_{BUT-55} \quad (1)$$

In Eq. (1), $E_{benzene@BUT-55}$ and E_{BUT-55} represent the total energies of benzene with and without BUT-55, respectively. $E_{benzene}$ is the energy of the benzene. According to this equation, a negative adsorption energy corresponds a stable adsorption structure.

For BUT-53, -54, and -58, the adsorption energy between benzene and the BUT substrate can also be calculated using the same equation with (1), respectively.

The DFT calculations are used for explanation of the benzene binding. Among these MOFs, BUT-53 and BUT-55 have similar binding energies, but their adsorption properties are different. the kinetics of benzene adsorption on BUT-53 and BUT-55 using thermogravimetric analysis (TGA) (Fig. S43b). These data reveal that benzene

is absorbed slightly slower by BUT-53 than by BUT-55. The adsorption performance of BUT-53 vs. BUT-55 can be described as follows: (1) At ultra-low pressure (the first few data points of the Henry's region), the isotherm of BUT-53 exhibited higher uptake and a stepper gradient than that of BUT-55 (Part 1 in Fig. S43a), indicative of stronger sorbent-sorbate binding for BUT-53 than BUT-55. The improved experimental sorption data are now consistent with the DFT calculations. (2) Between the crossing and plateau (Part 2 in Fig. S43a), BUT-55 outperformed BUT-53 and exhibited higher uptake. (3) Both isotherms exhibited a plateau after reaching maximum uptakes (Part 3 in Fig. S43a) defined by the specific surface area of the respective MOFs.

In addition, the SCXRD data reveal that a minor ligand swing was observed before benzene adsorption in BUT-53, which was immobilized by benzene adsorption (Fig. S43c). This guest induced structural immobilization has not been observed in the other BUT sorbents. This may also contribute to their different adsorption performances.

Section 8. Additional Figures and Tables

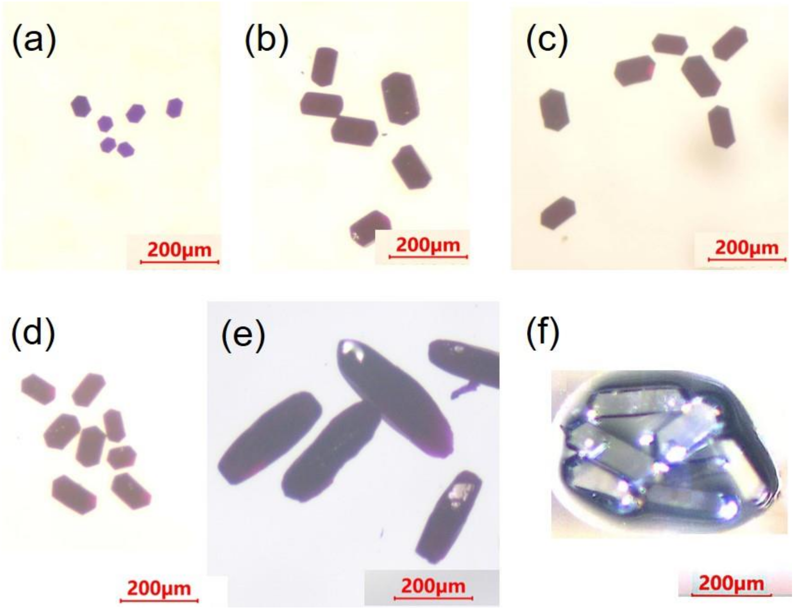

Fig. S1. Crystal images of as-synthesized (a) BUT-53, (b) BUT-54, (c) BUT-55, (d) BUT-56, (e) BUT-57, (f) BUT-58.

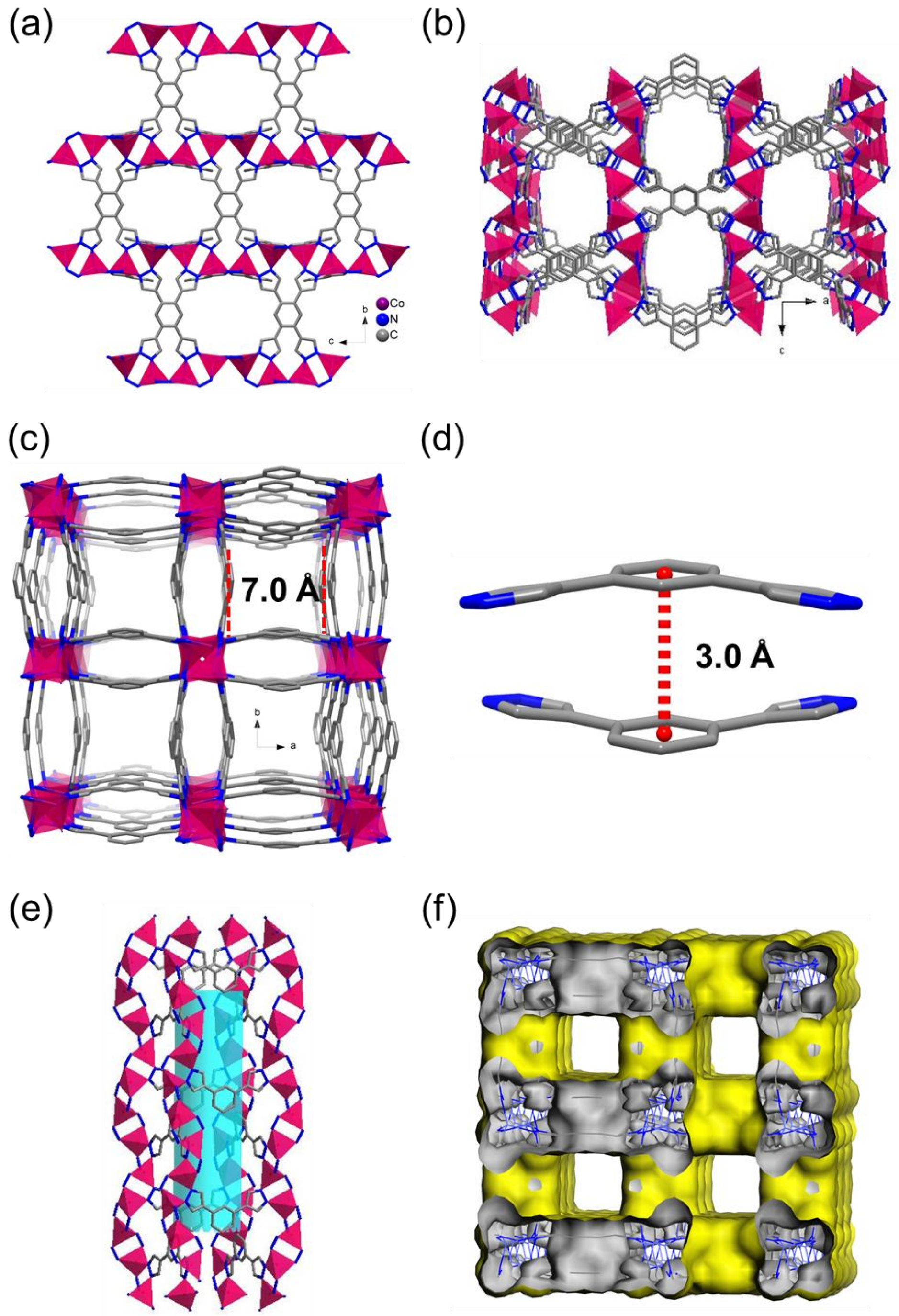

Fig. S2. Crystal structure of BUT-53 as viewed along the crystallographic (a) a, (b) b, and (c) c axis. (d) Ligand stacking and (e) single-walled structure in BUT-53. (f) Internal structure of pores in BUT-53 illustrated by the Connolly surface in yellow.

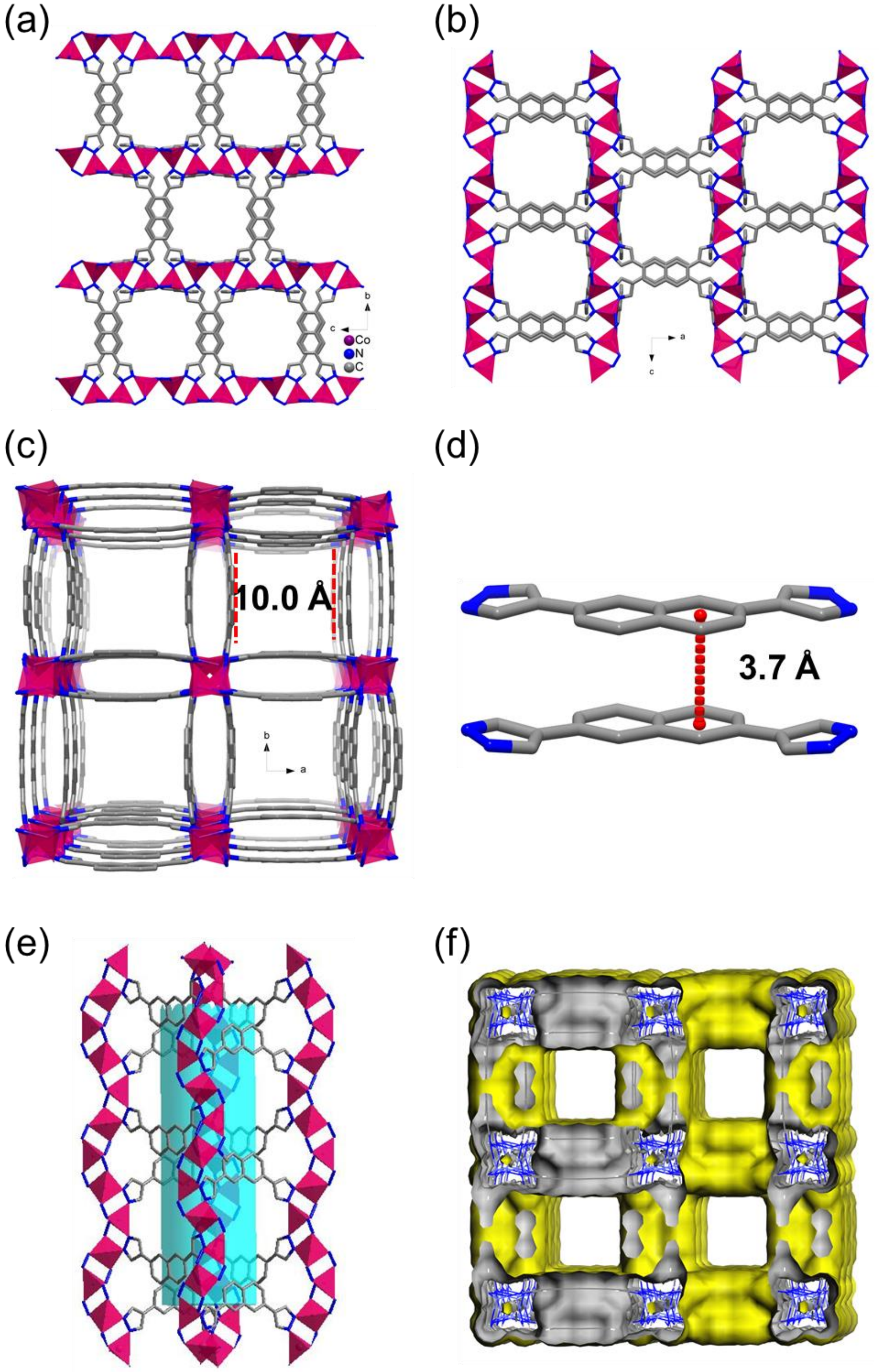

Fig. S3. Crystal structure of BUT-54 as viewed along the crystallographic (a) a, (b) b, and (c) c axis. (d) Ligand stacking and (e) single-walled structure in BUT-54. (f) Internal structure of pores in BUT-54 illustrated by the Connolly surface in yellow.

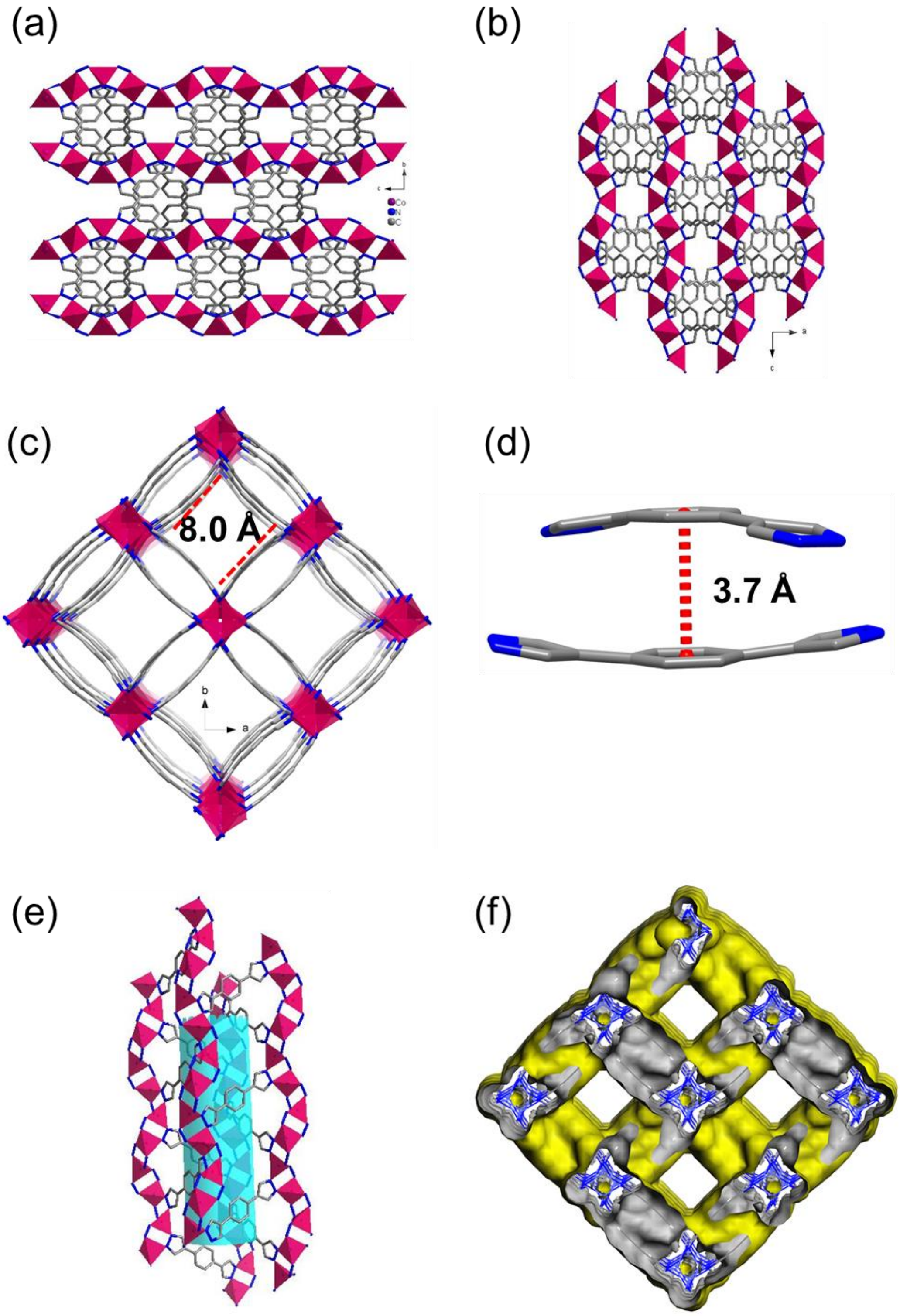

Fig. S4. Crystal structure of BUT-55 as viewed along the crystallographic (a) a, (b) b, and (c) c axis. (d) Ligand stacking and (e) single-walled structure in BUT-55. (f) Internal structure of pores in BUT-55 illustrated by the Connolly surface in yellow.

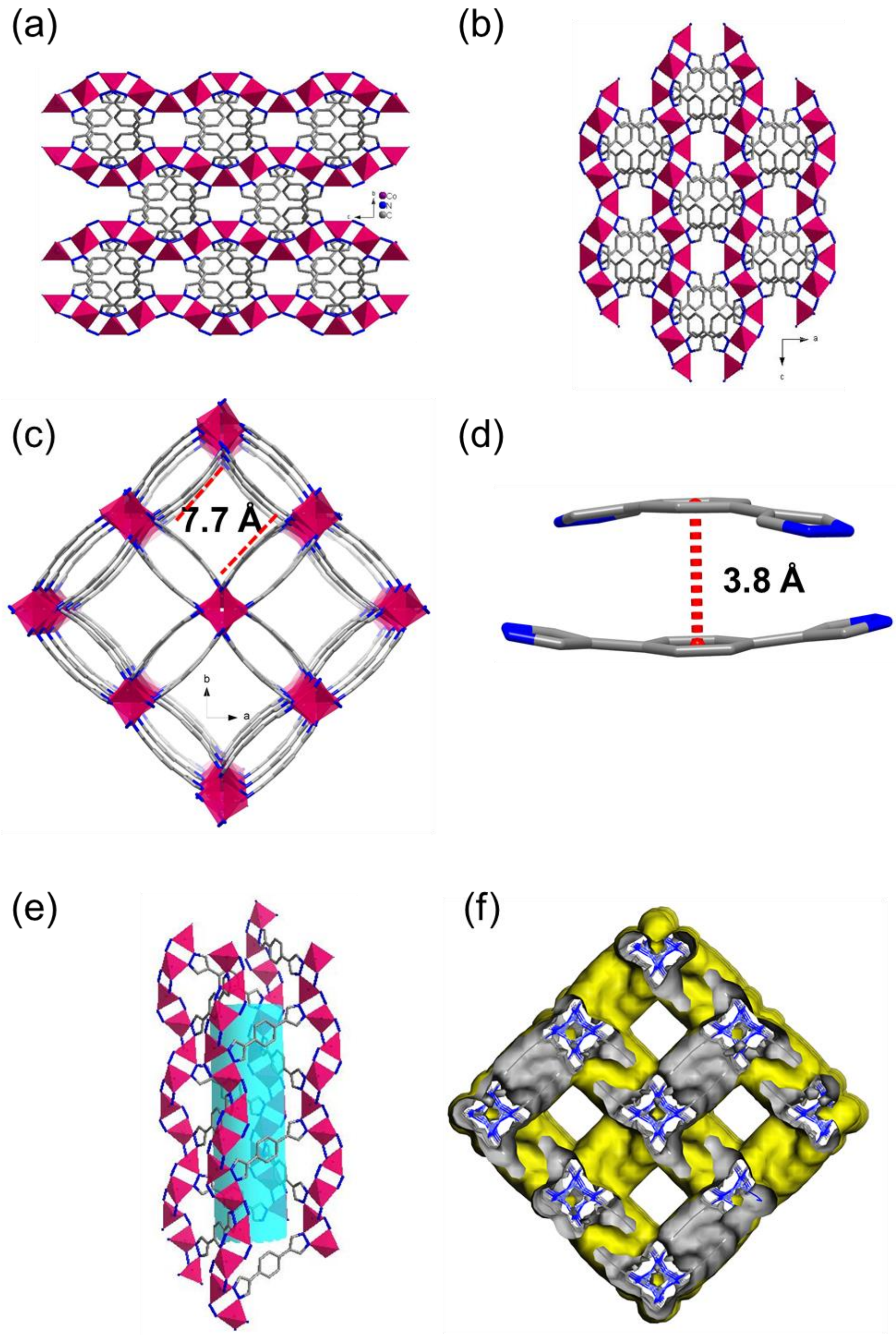

Fig. S5. Crystal structure of BUT-56 as viewed along the crystallographic (a) a, (b) b, and (c) c axis. (d) Ligand stacking and (e) single-walled structure in BUT-56. (f) Internal structure of pores in BUT-56 illustrated by the Connolly surface in yellow.

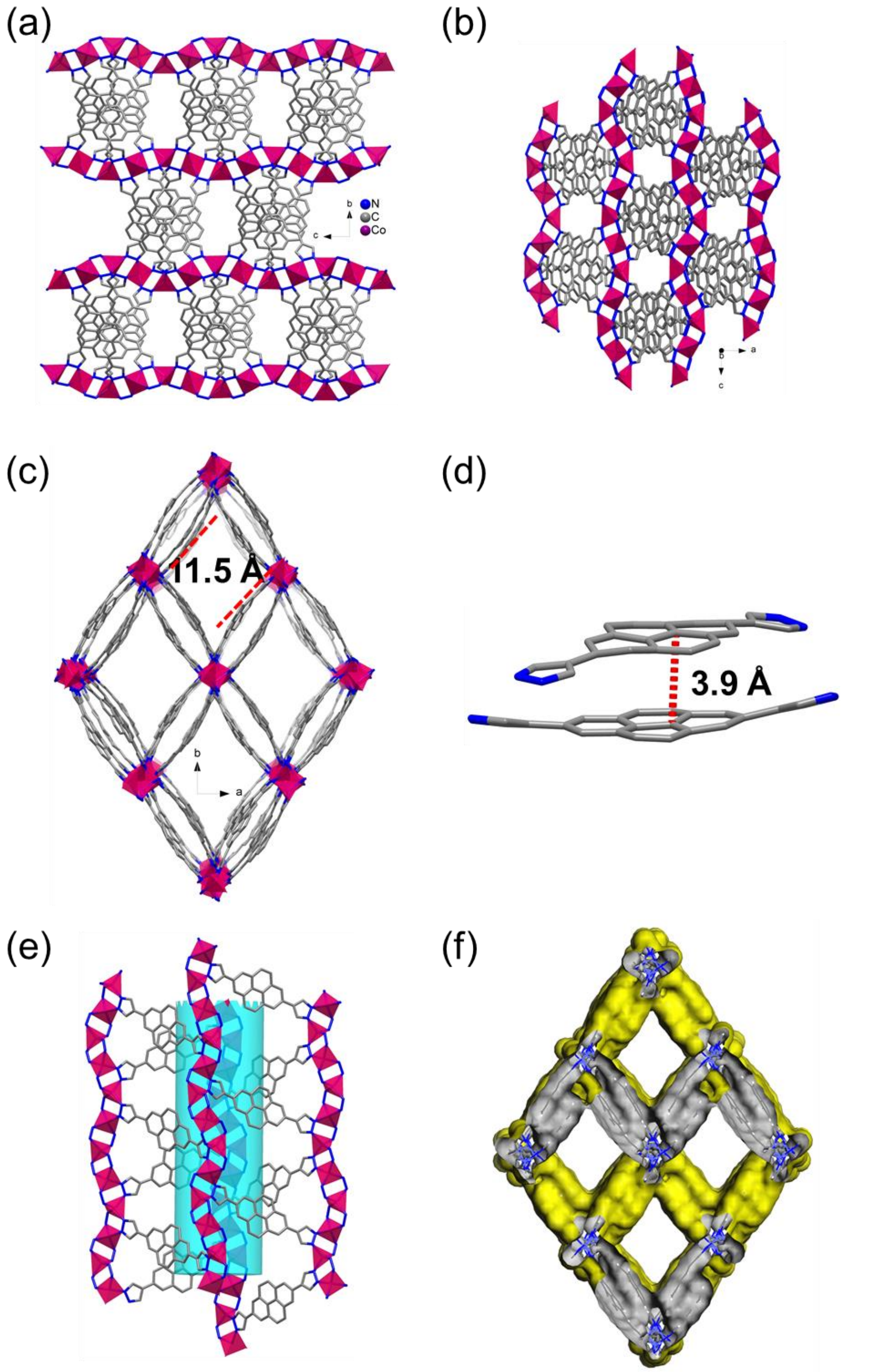

Fig. S6. Crystal structure of BUT-57 as viewed along the crystallographic (a) a, (b) b, and (c) c axis. (d) Ligand stacking and (e) single-walled structure in BUT-57. (f) Internal structure of pores in BUT-57 illustrated by the Connolly surface in yellow.

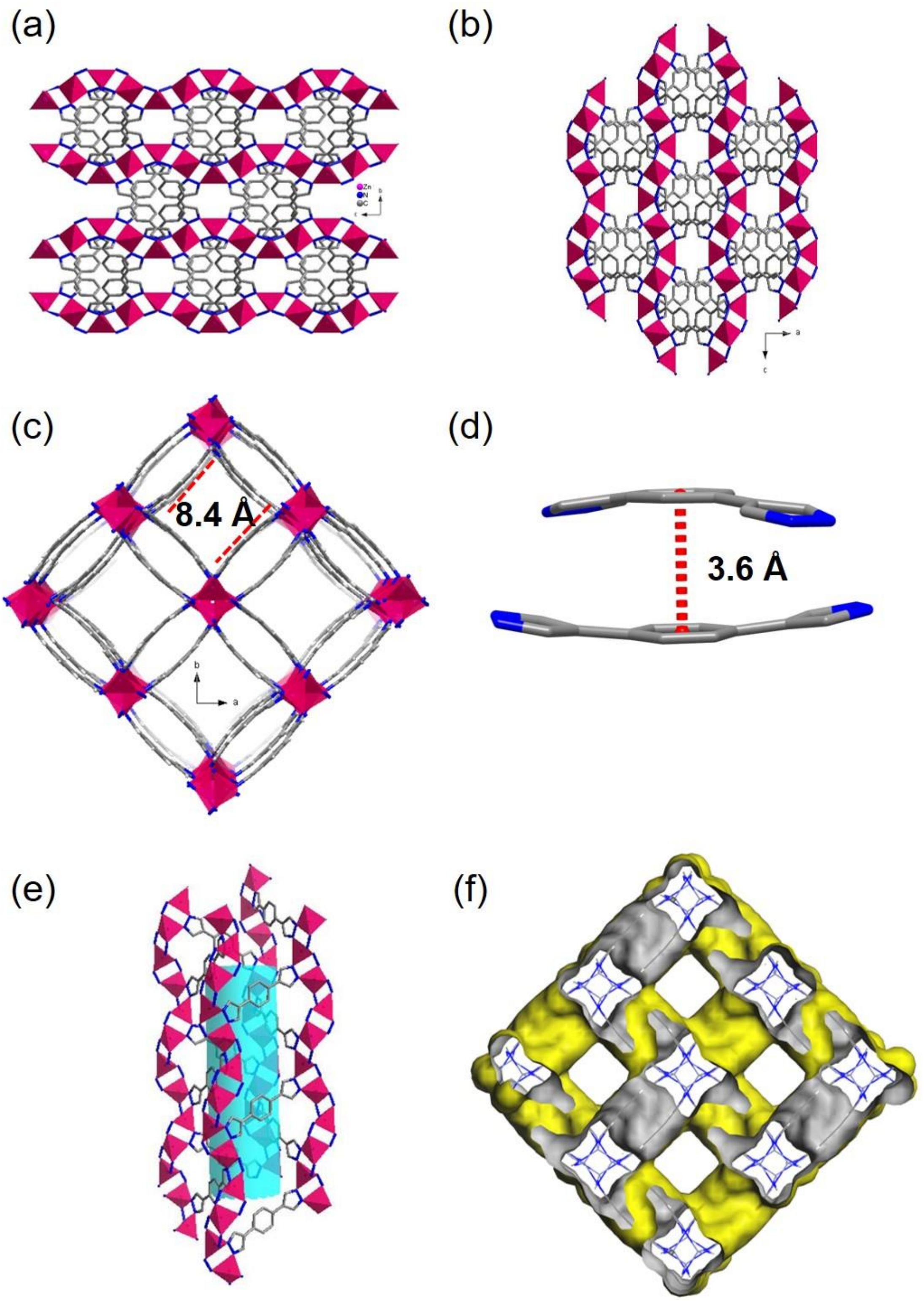

Fig. S7. Crystal structure of BUT-58 as viewed along the crystallographic (a) a, (b) b, and (c) c axis. (d) Ligand stacking and (e) single-walled structure in BUT-58. (f) Internal structure of pores in BUT-58 illustrated by the Connolly surface in yellow.

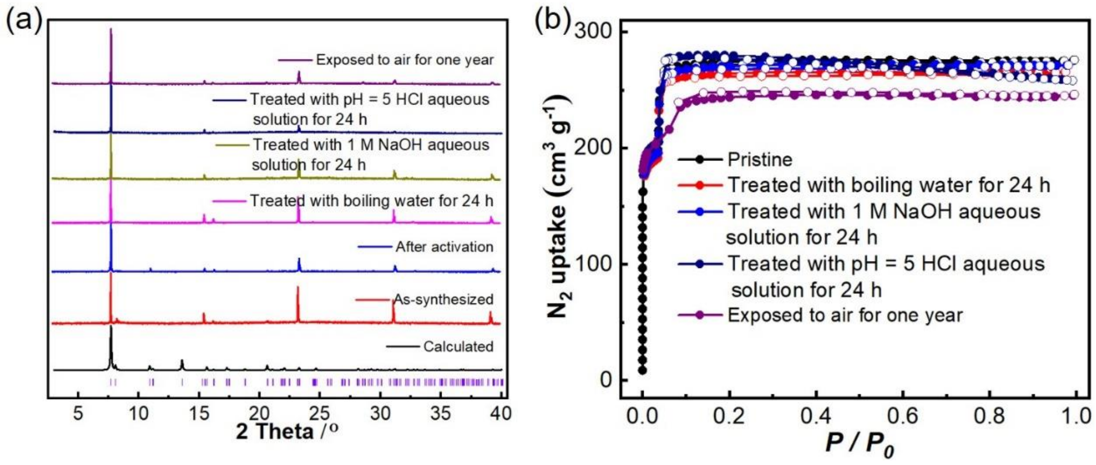

Fig. S8. (a) PXRD patterns of BUT-53 (calculated, as-synthesized, and exposed samples). (b) N_2 adsorption/desorption isotherms at 77 K for BUT-53 after various stress tests.

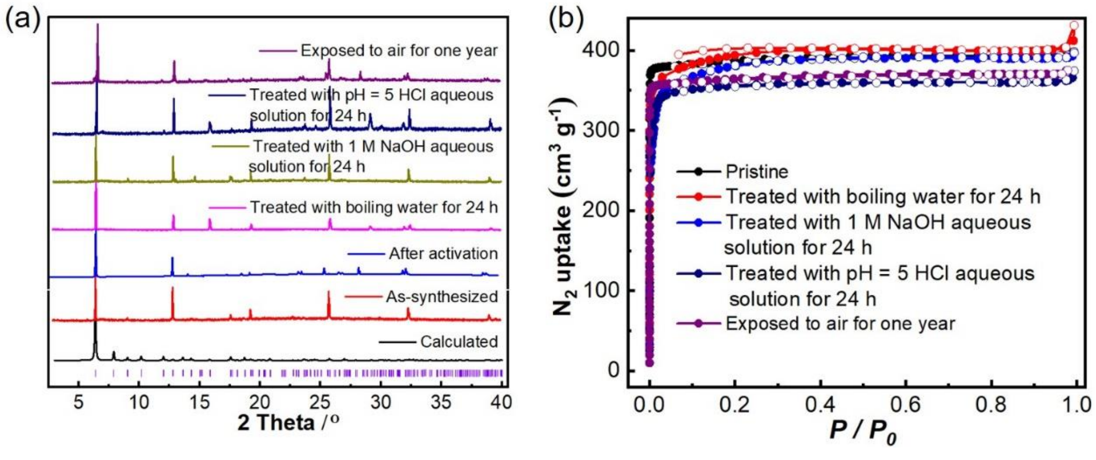

Fig. S9. (a) PXRD patterns of BUT-54 (calculated, as-synthesized, and exposed samples). (b) N_2 adsorption/desorption isotherms at 77 K for BUT-54 after various stress tests.

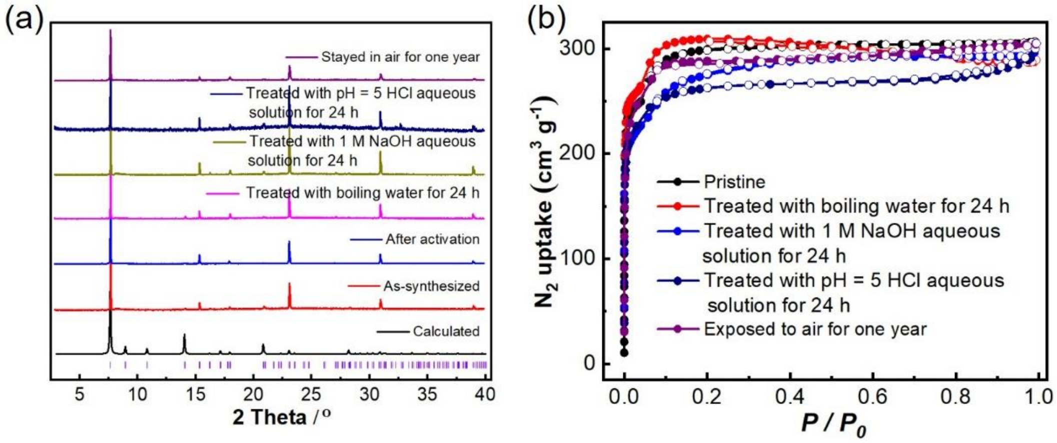

Fig. S10. (a) PXRD patterns of BUT-55 (calculated, as-synthesized, and exposed samples). (b) N_2 adsorption/desorption isotherms at 77 K for BUT-55 after various stress tests.

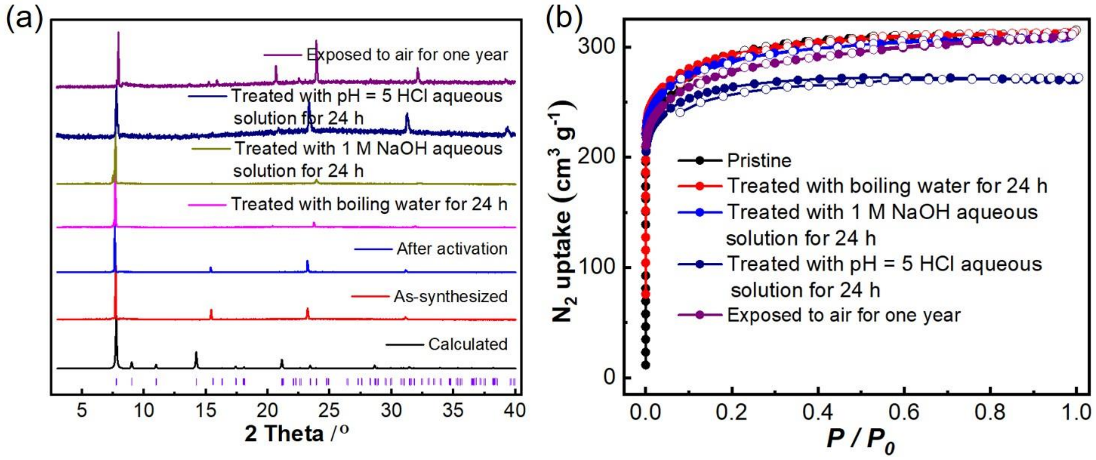

Fig. S11. (a) PXRD diffractograms of BUT-56 (calculated, as-synthesized, and exposed samples). (b) N_2 adsorption/desorption isotherms at 77 K for BUT-56 after stress tests.

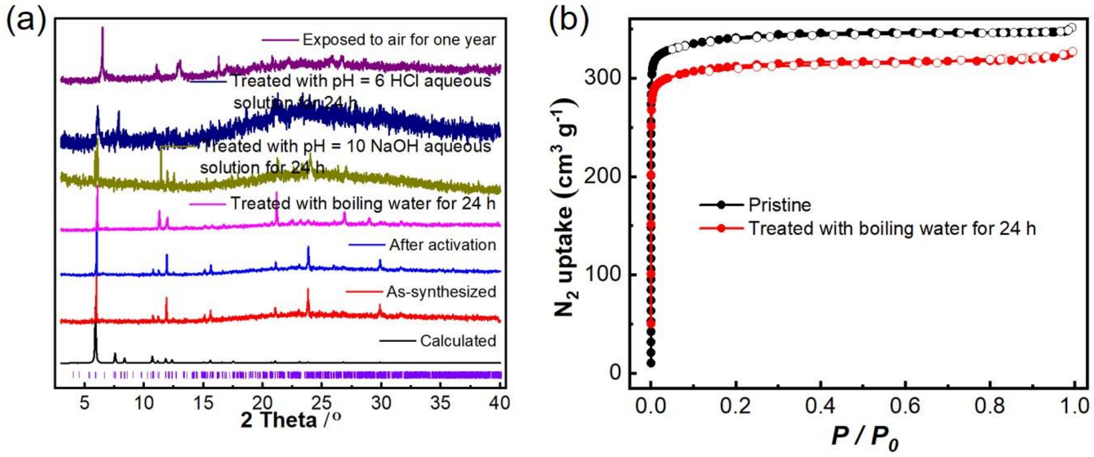

Fig. S12. (a) PXRD diffractograms of BUT-57 (calculated, as-synthesized, and exposed samples). (b) N_2 adsorption/desorption isotherms at 77 K for BUT-57 after stress tests.

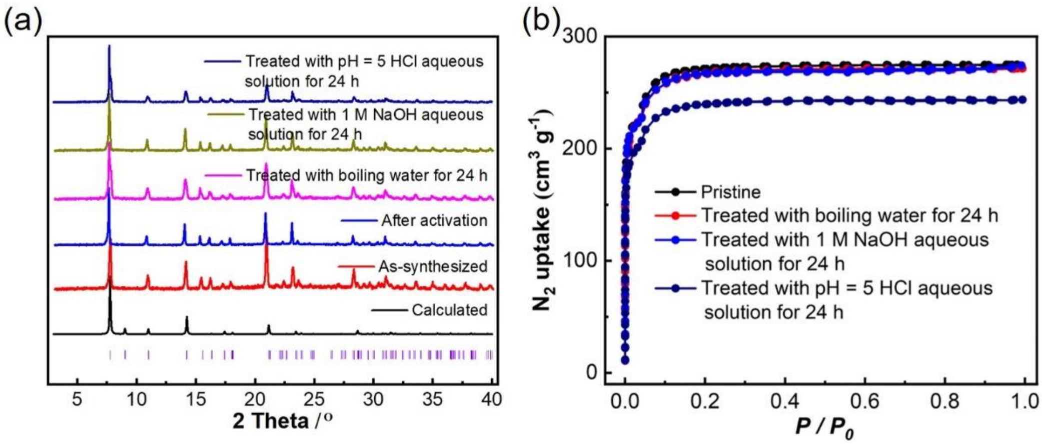

Fig. S13. (a) PXRD diffractograms of BUT-58 (calculated, as-synthesized, and exposed samples). (b) N_2 adsorption/desorption isotherms at 77 K for BUT-58 after stress tests.

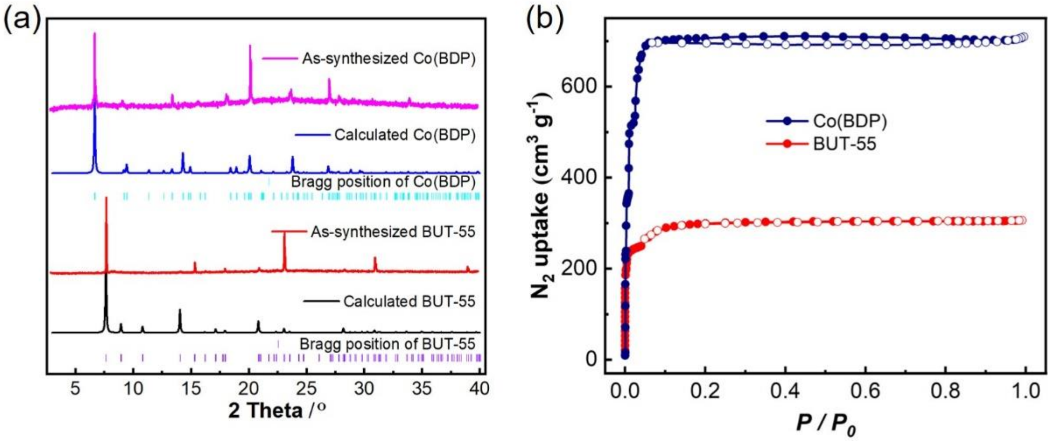

Fig. S14. Comparison of (a) PXRD patterns and (b) N_2 adsorption/desorption isotherms of BUT-55 and Co(BDP).

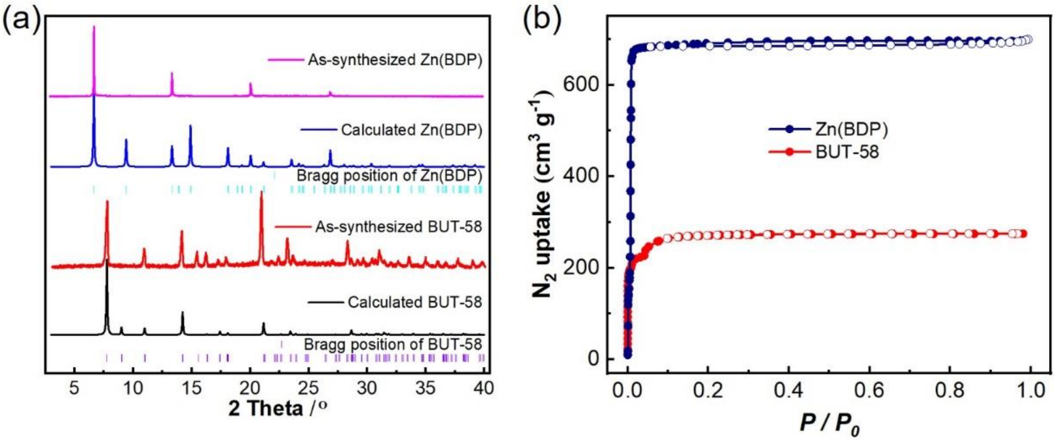

Fig. S15. Comparison of (a) PXRD patterns and (b) N_2 adsorption/desorption isotherms of BUT-58 and Zn(BDP).

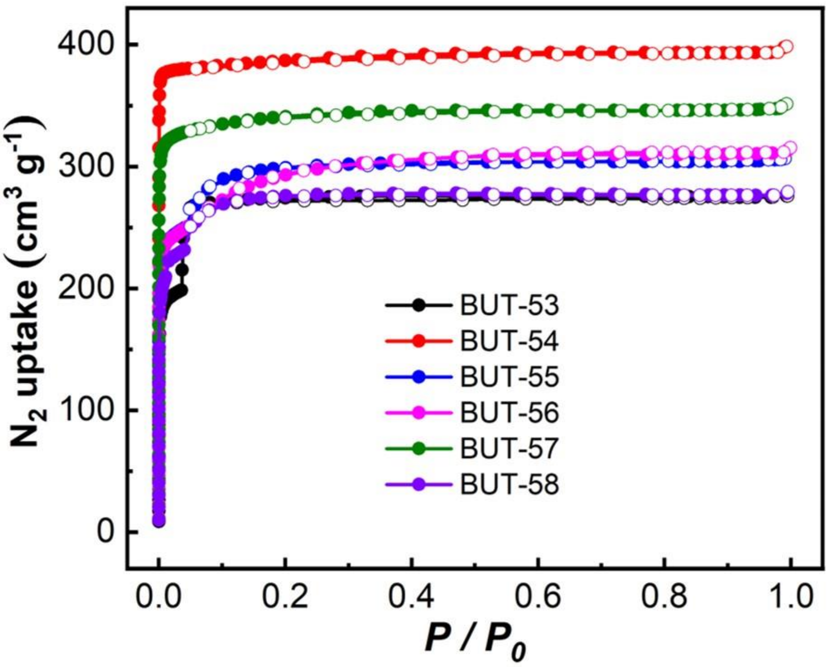

Fig. S16. N_2 adsorption/desorption isotherms measured at 77 K for activated BUT-53 to BUT-58 samples.

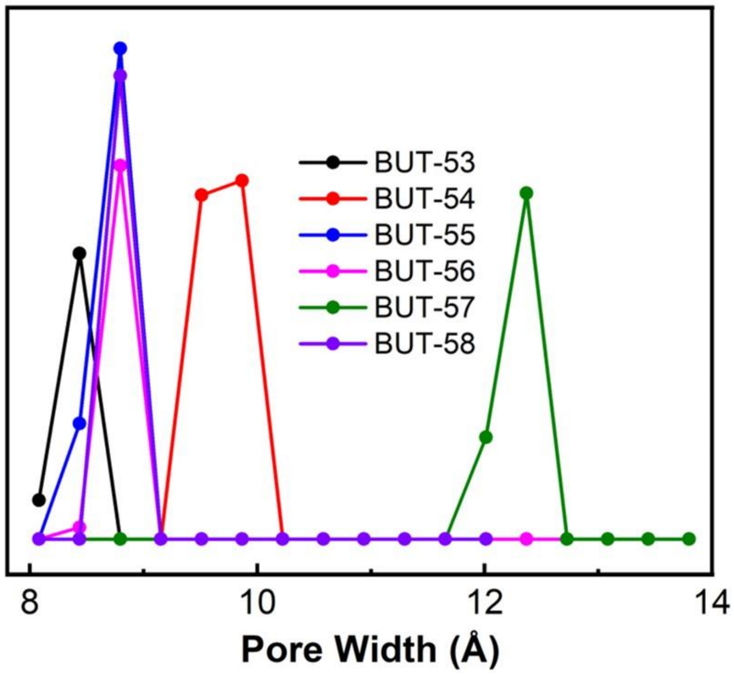

Fig. S17. DFT pore size distributions in BUT-53 to BUT-58.

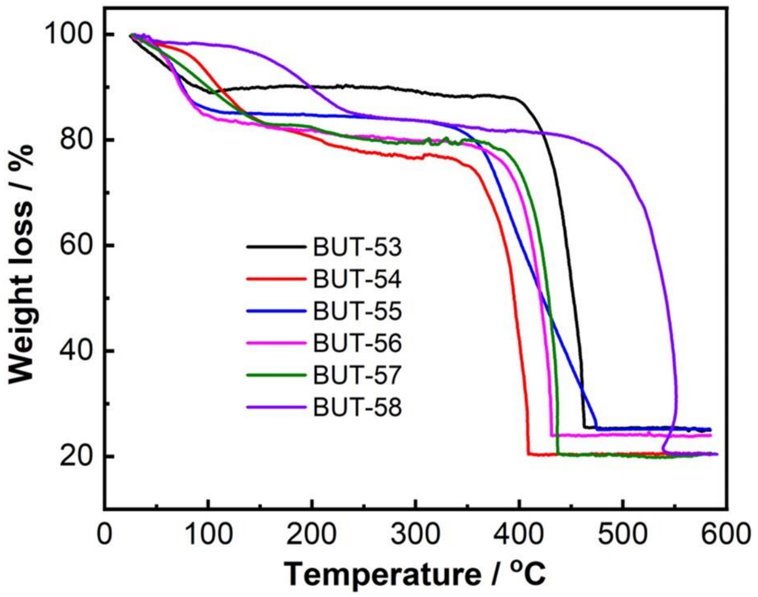

Fig. S18. TGA curves for BUT-53 to BUT-58.

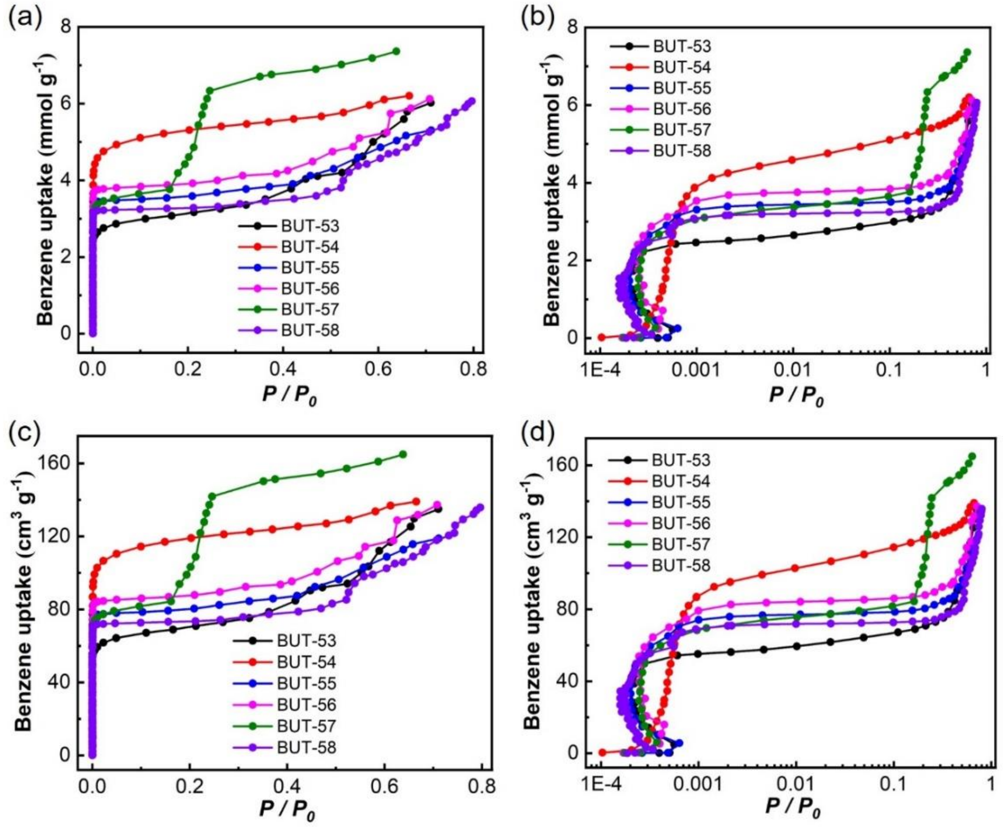

Fig. S19. Benzene adsorption isotherms of BUT-53 to BUT-58 collected at equilibrium conditions of 100 s/0.01% on Micrometrics ASAP 2020 at 298 K.

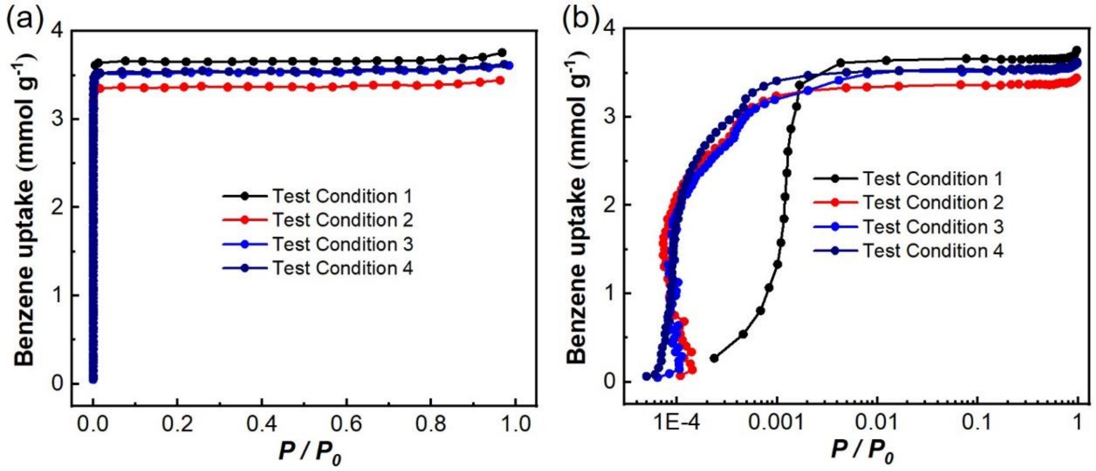

Fig. 20. Benzene adsorption for BUT-55 collected on a BELSORP-maxII instrument at 298 K. Conditions 1-4 correspond to equilibration parameters of 300 s/0.3%, 300 s/0.1%, 600 s/0.1%, and 1000 s/0.1%, respectively.

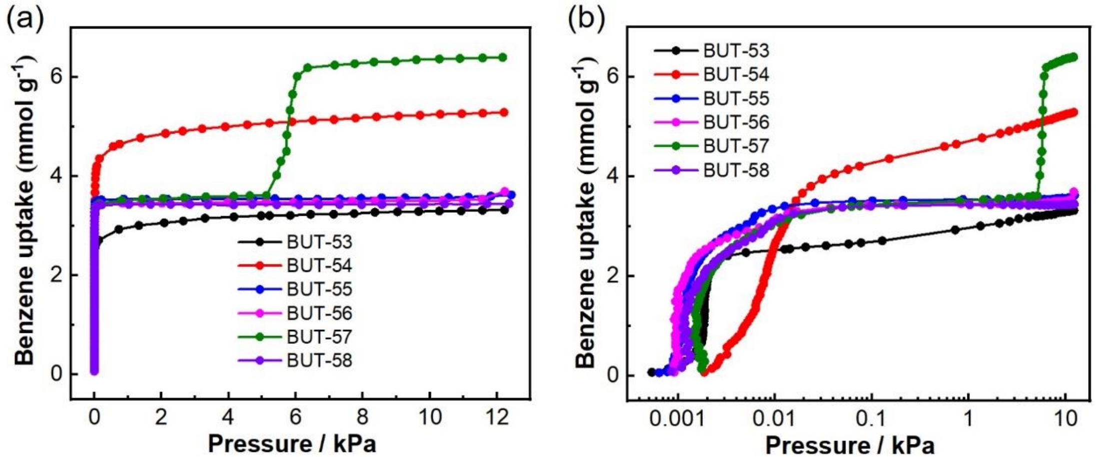

Fig. S21. (a, b) Benzene adsorption isotherms of BUT-53-58 collected at equilibrium conditions of 1000 s/0.1% on BELSORP-maxII.

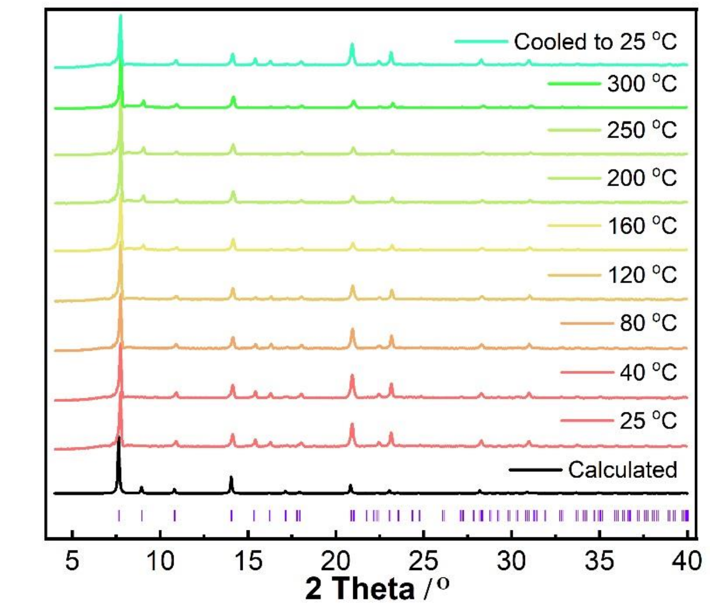

Fig. S22. *In situ* variable temperature PXRD patterns of BUT-55 with DMF.

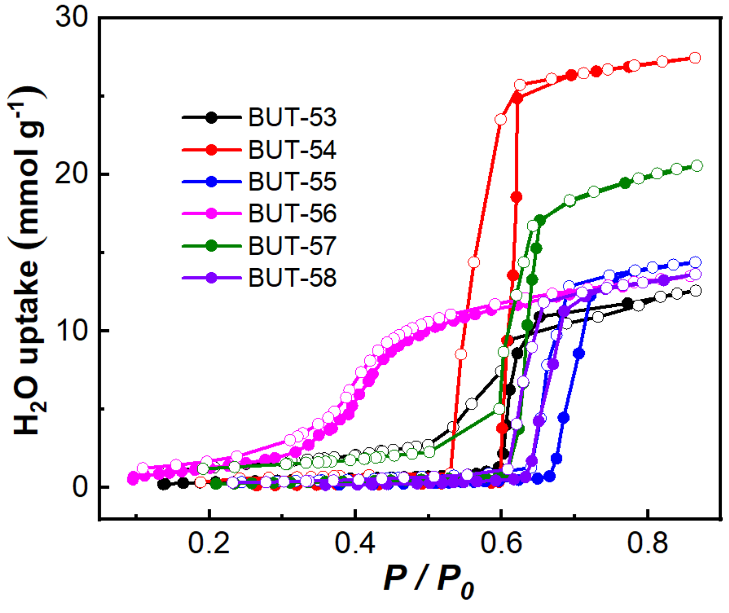

Fig. S23. Water vapor adsorption/desorption isotherms for BUT-53 to BUT-58 at 298 K.

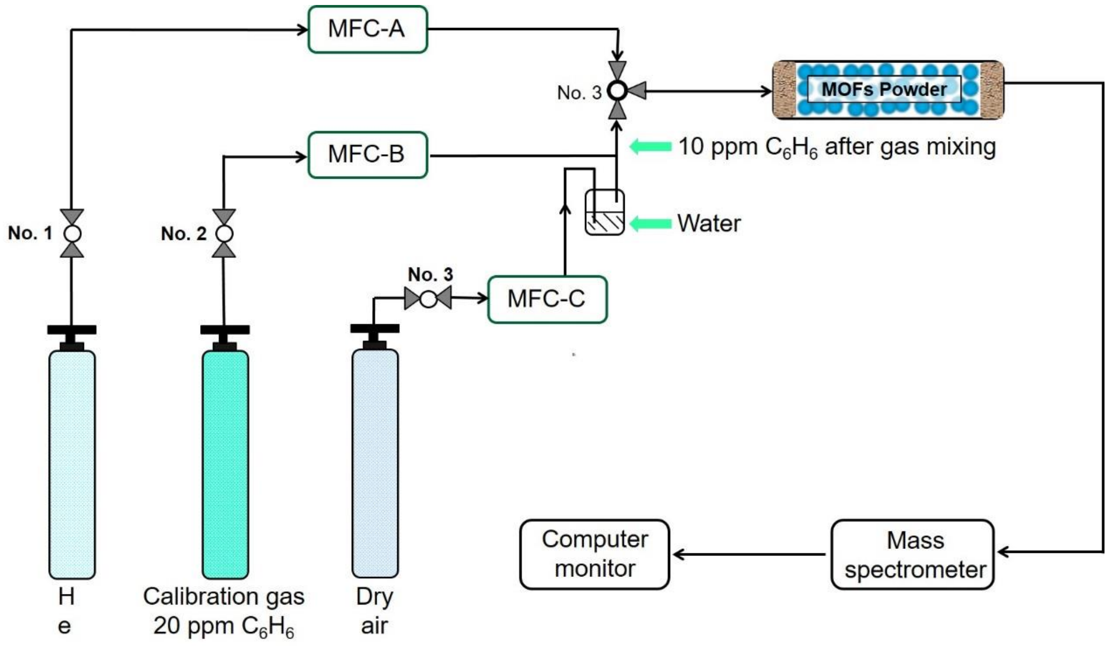

Fig. S24. Schematic illustration of the apparatus used for breakthrough experiments.

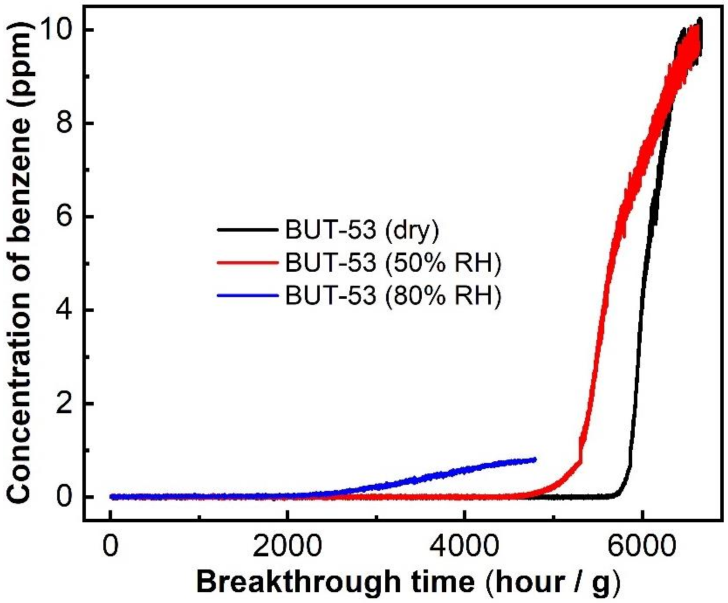

Fig. S25. Benzene breakthrough curves collected under dry conditions, RH 50% and 80% for BUT-53 at 298 K.

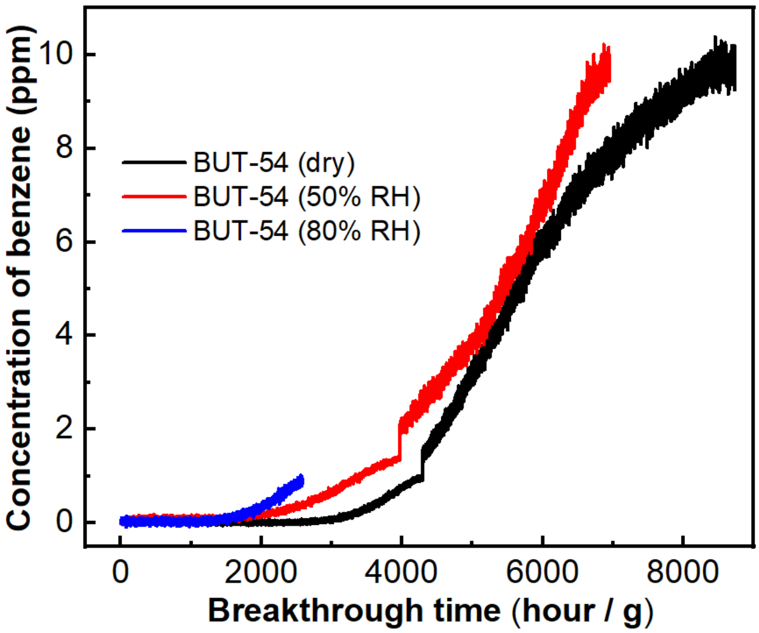

Fig. S26. Benzene breakthrough curves collected under dry conditions, RH 50% and 80% for BUT-54 at 298 K.

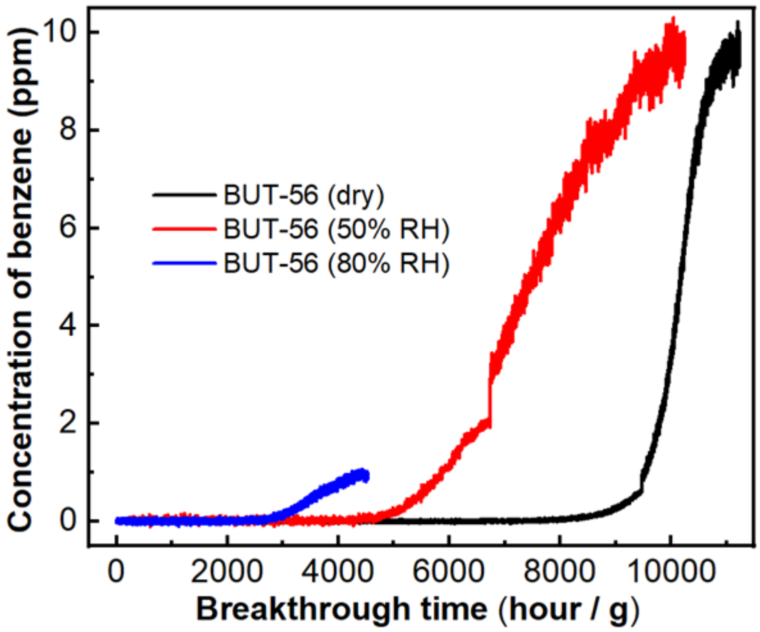

Fig. S27. Benzene breakthrough curves collected under dry conditions, RH 50% and 80% for BUT-56 at 298 K.

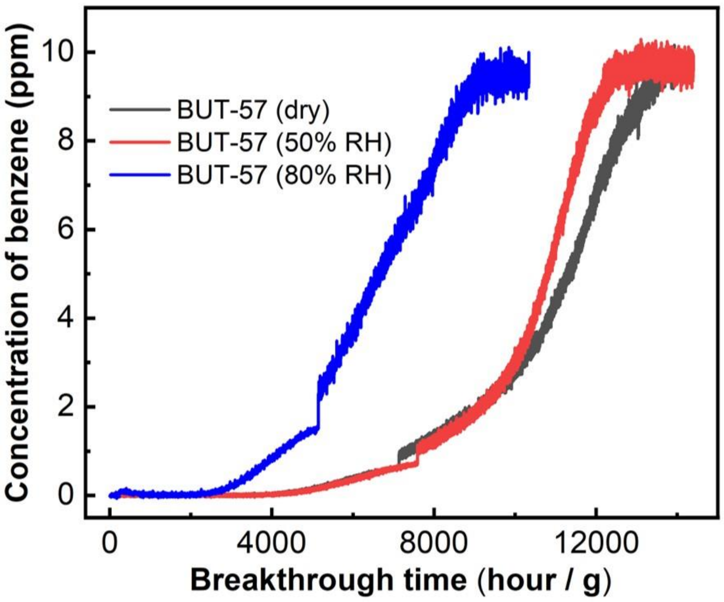

Fig. S28. Benzene breakthrough curves collected under dry conditions, RH 50% and 80% for BUT-57 at 298 K.

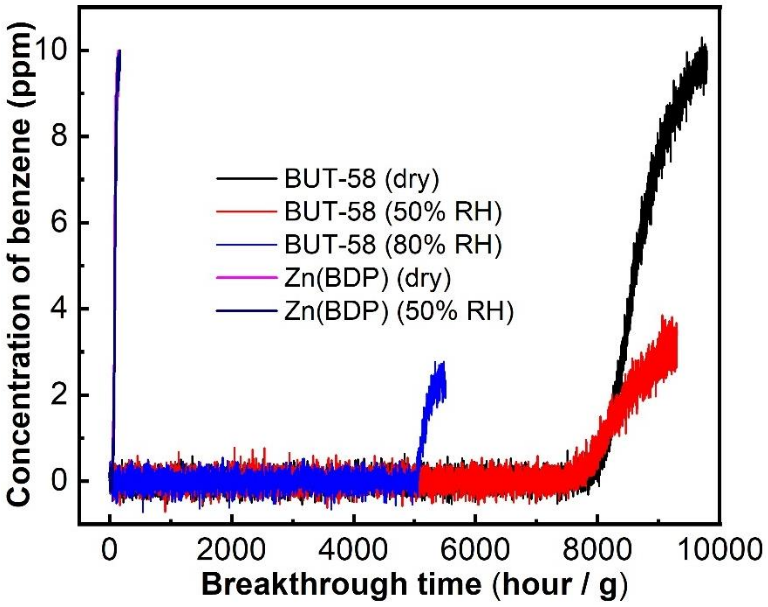

Fig. S29. Benzene breakthrough curves collected under dry conditions, RH 50% and 80% for BUT-58 at 298 K.

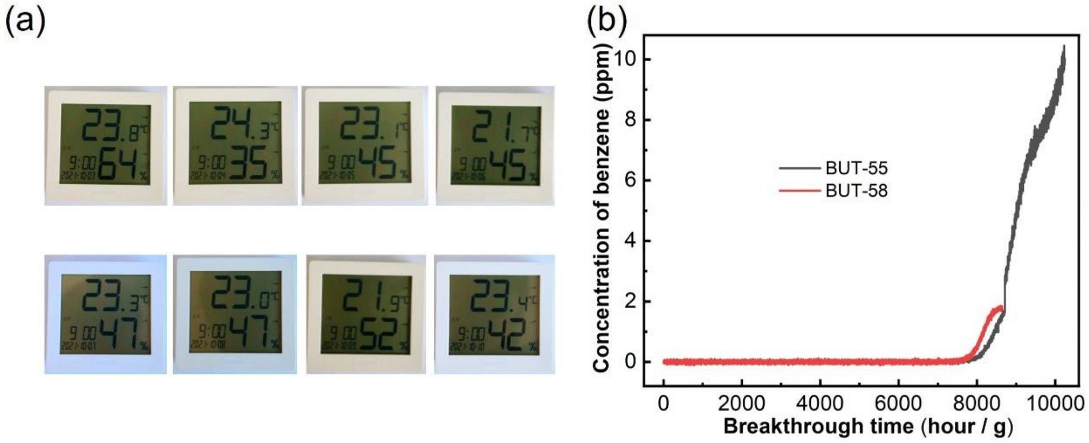

Fig. S30. (a) Display screen of a humidity sensor showing real-time RH (ranging from 35 to 64%, at 9 am from 3rd to 10th Oct in Beijing). (b) Benzene breakthrough curve collected on BUT-55 and BUT-58 samples exposed to moisture for one week before testing.

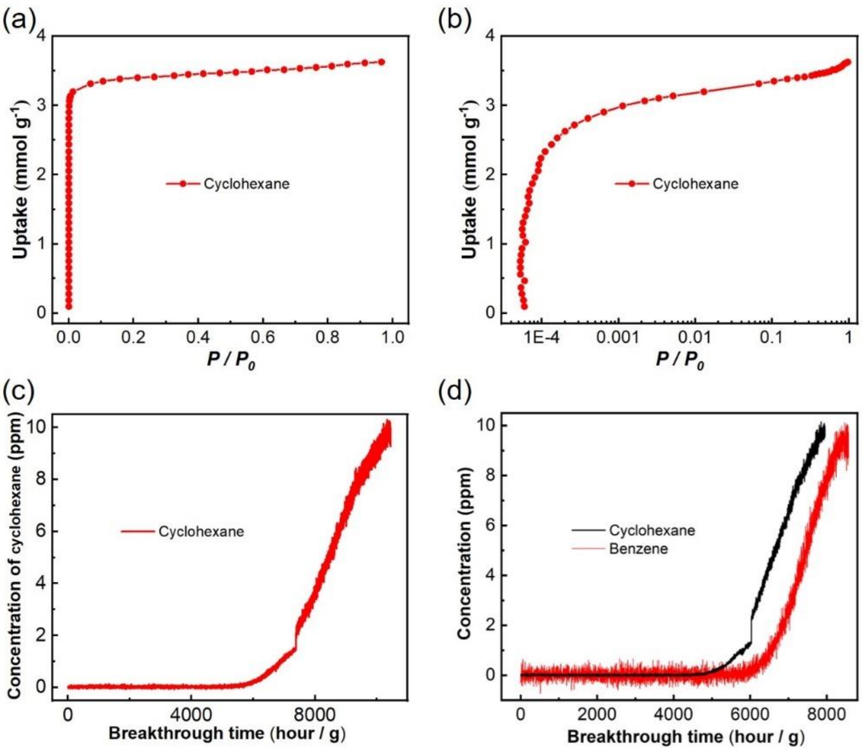

Fig. S31. Adsorption isotherms and breakthrough curves for BUT-55 at 298 K: (a) cyclohexane adsorption isotherm; (b) logarithmic scale plot of P/P_0; (c) cyclohexane breakthrough curve; (d) competitive breakthrough curve for benzene/cyclohexane.

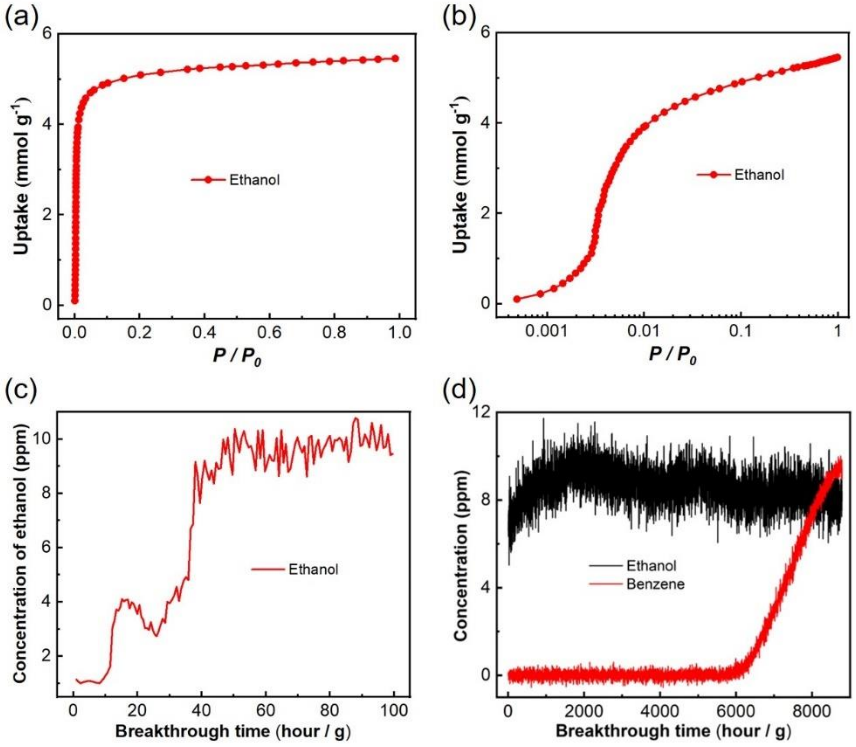

Fig. S32. Adsorption isotherms and breakthrough curves for BUT-55 at 298 K: (a) ethanol adsorption isotherm;(b) logarithmic scale plot of P/P_0; (c) ethanol breakthrough curve’ (d) competitive breakthrough curve for benzene/ethanol.

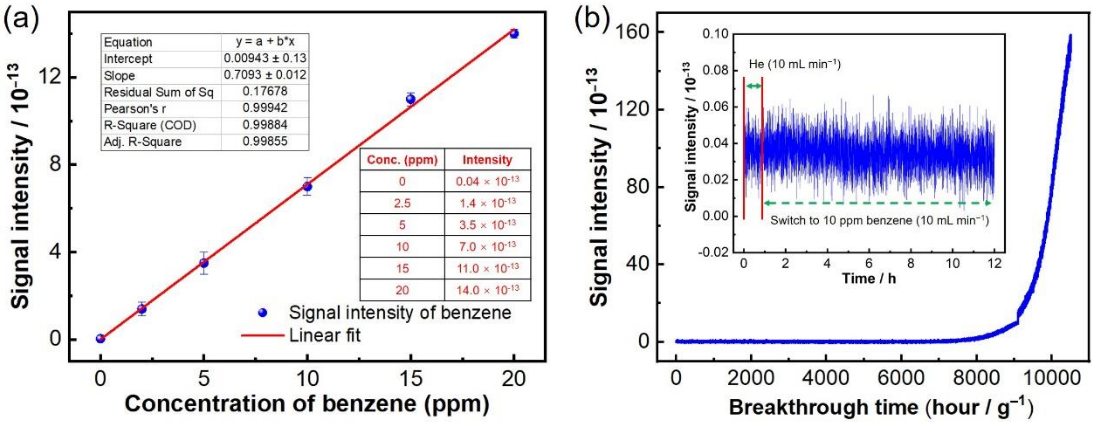

Fig. S33. (a) Standard curve of concentration of benzene. (b) Breakthrough curves for BUT-55 with 10 ppm benzene at 298 K (inset: the breakthrough curve for the gas was changed from He to the 10 ppm benzene gas mixture at the 30th minute).

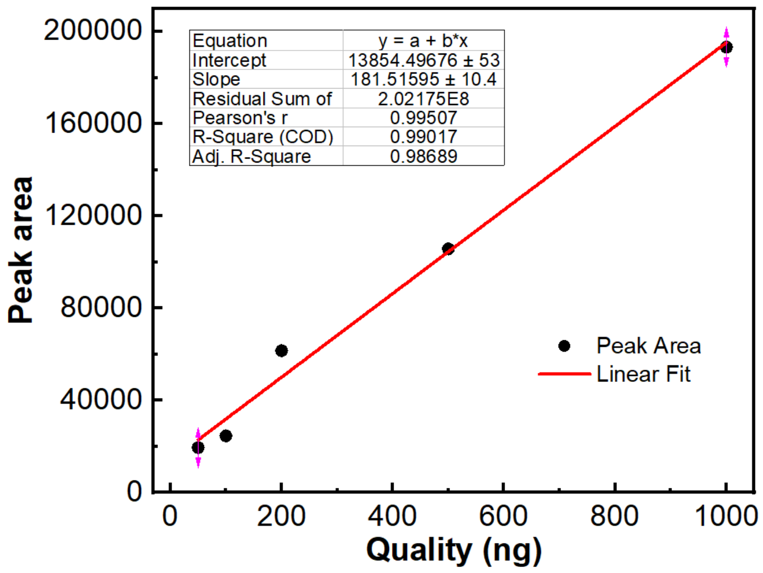

Fig. S34. Standard curve for benzene concentration detection by GC-MS.

Table S2. Experiments for determining the LOD for benzene in GC-MS.

Peak area	32332	32205	32096	31751	31733	31588	32205
Measured level (ng)	101.8	101.1	100.5	98.6	98.5	97.7	101.1
Standard deviation	1.599						
LOD	5.02 ng						

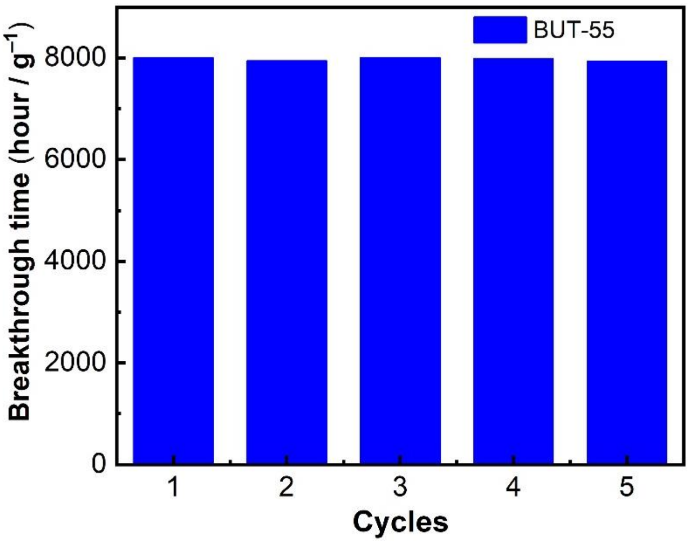

Fig. S35. Breakthrough experiments conducted on BUT-55 under dry conditions at 298 K over five consecutive cycles.

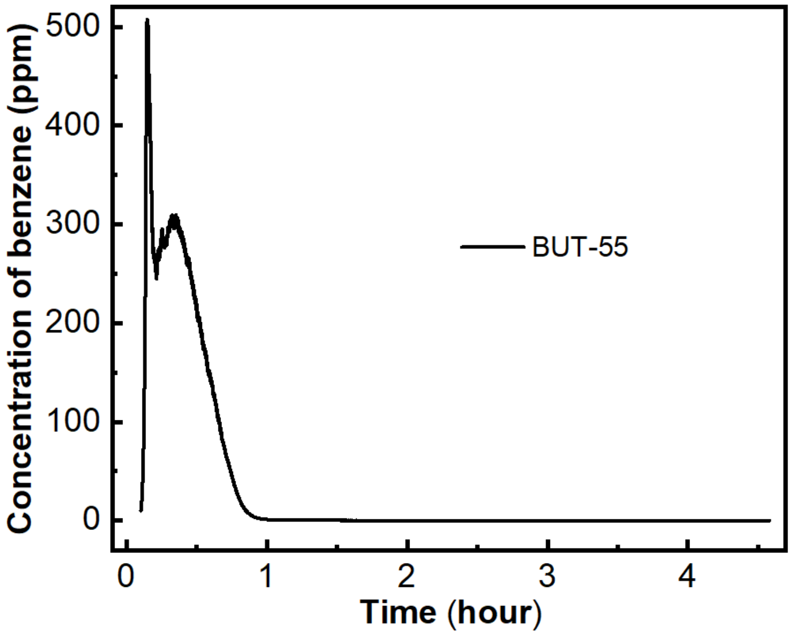

Fig. S36. Benzene desorption curve recorded on the BUT-55 packed column at 120 ^{o}C with a N_2 flow of 20 mL/min.

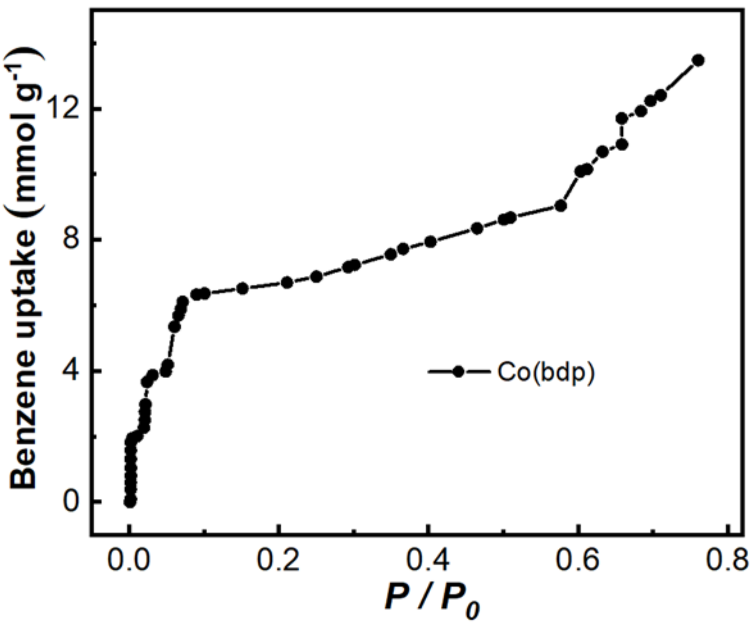

Fig. S37. Benzene vapor adsorption isotherm for Co(BDP) at 298 K.

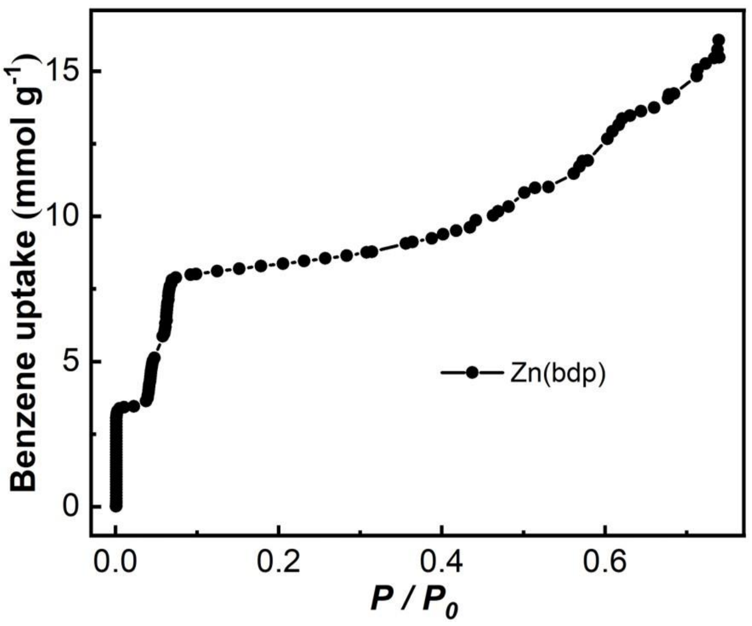

Fig. S38. Benzene vapor adsorption isotherm for Zn(BDP) at 298 K.

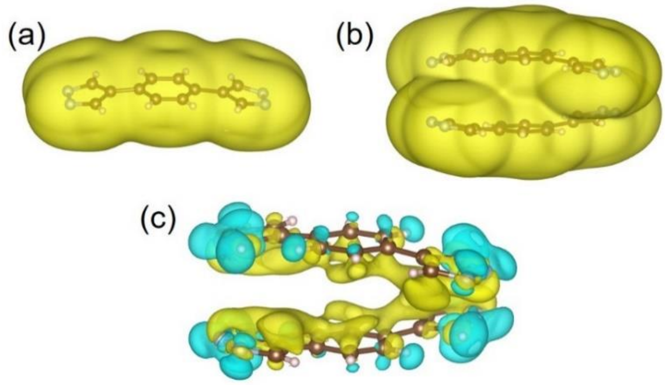

Fig. S39. Electronic cloud density of (a) single-layered BDP ligands in Co(BDP) and (b) double-layered BDP ligands in BUT-55. (c) Isosurfaces of charge density difference for the interaction of double-layered ligands in BUT-55. Yellow indicates electronic accumulation, and light blue indicates depletion.

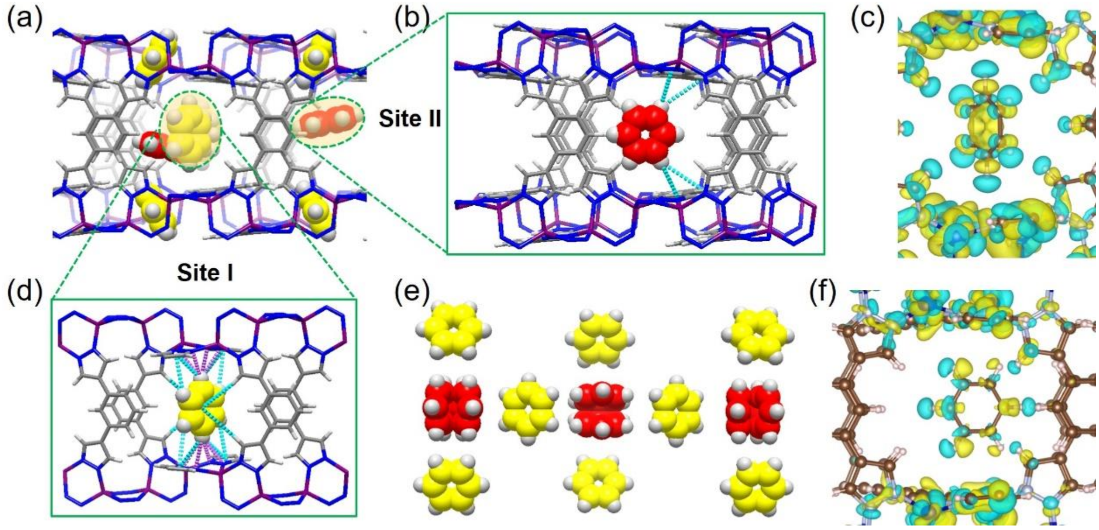

Fig. S40. SCXRD structure of C_6H_6@BUT-53. (a) Adsorbed benzene molecules in pores as viewed along the c axis. Binding of adsorbed benzene molecules to the framework at site I (b) and site II (d). (e) Packing of two types of adsorbed benzene molecules. Isosurfaces of charge density difference for the interaction of (c) site I and (f) site II with adsorbed benzene molecules in BUT-53. Yellow indicates electronic accumulation, and light blue indicates depletion.

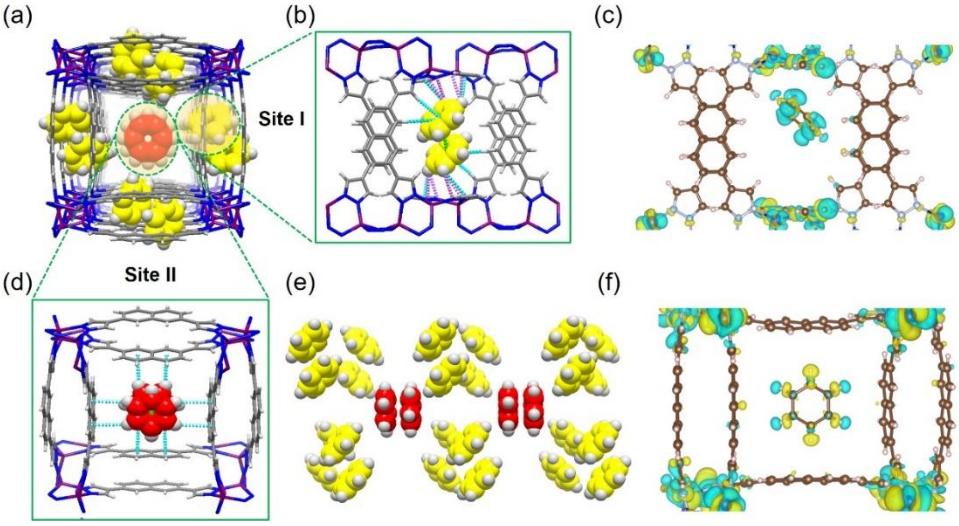

Fig. S41. SCXRD structure of C_6H_6@BUT-54. (a) Adsorbed benzene molecules in pores as viewed along the c axis. Binding of adsorbed benzene molecules to the framework at site I (b) and site II (d). (e) Packing of two types of adsorbed benzene molecules. Isosurfaces of charge density difference for the interaction of (c) site I and (f) site II with adsorbed benzene molecules in BUT-54. Yellow indicates electronic accumulation, and light blue indicates depletion.

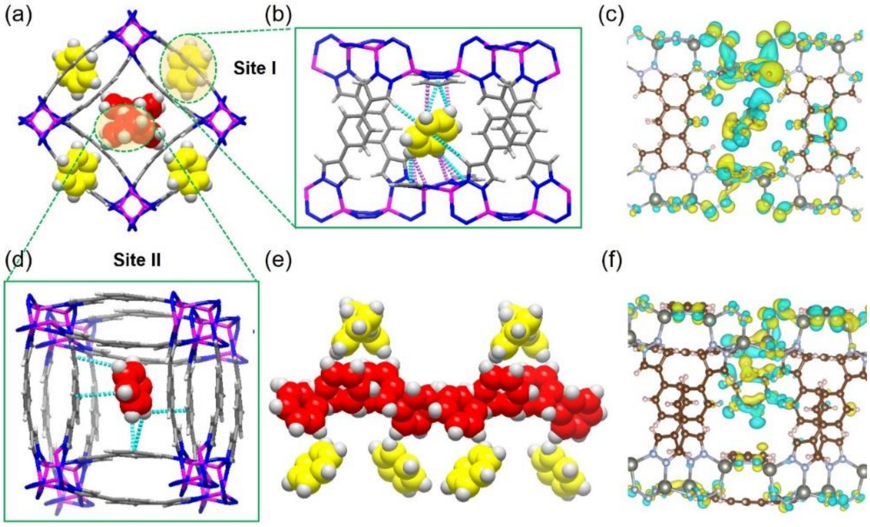

Fig. S42. SCXRD structure of C_6H_6@BUT-58. (a) Adsorbed benzene molecules in pores as viewed along the c axis. Binding of adsorbed benzene molecules to the framework at site I (b) and site II (d). (e) Packing of two types of adsorbed benzene molecules. Isosurfaces of charge density difference for the interaction of (c) site I and (f) site II with adsorbed benzene molecules in BUT-58. Yellow indicates electronic accumulation, and light blue indicates depletion.

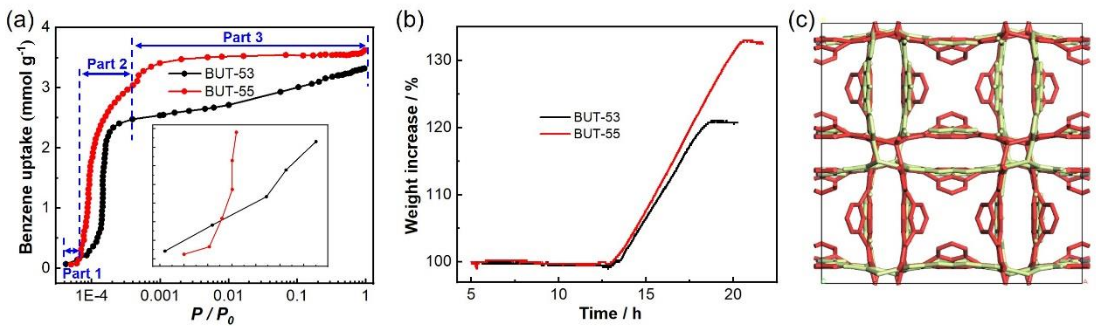

Fig. S43. (a) Logarithmic scale plot of P/P_0 to view benzene adsorption of BUT-53 and BUT-55 (insert: enlarged view of Part 1). (b) TGA curves of benzene adsorption by BUT-53 and BUT-55 (gas flow of 50 mL/min and benzene concentration of 800 ppm). (c) Ligand swing (between positions of the red and yellow) before benzene adsorption, and the immobilized ligand position (yellow) after benzene adsorption in BUT-53 observed from the SCXRD structures.

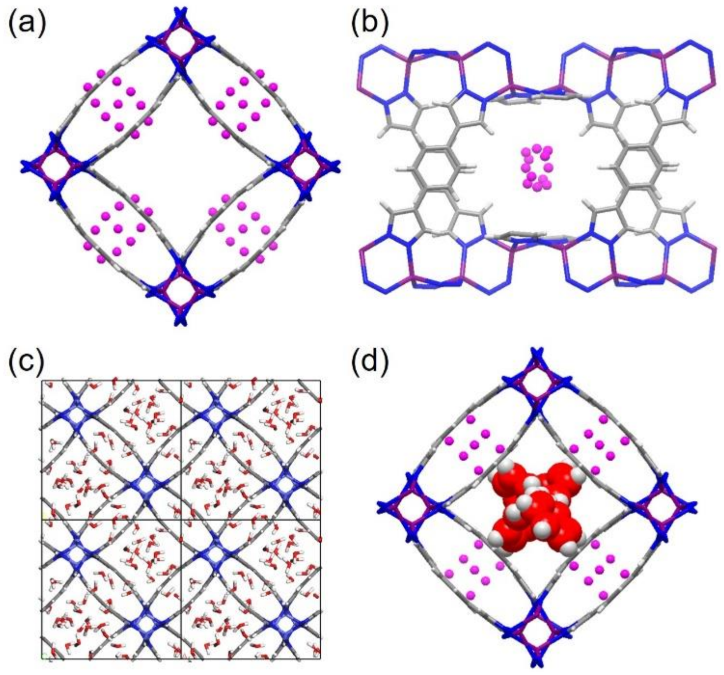

Fig. S44. Single crystal structure of H_2O@BUT-55 viewed along the (a) c and (d) b axis; (c) Simulated distribution of adsorbed water molecules in the unit cell of BUT-55; (d) C_6H_6/H_2O@BUT-55 viewed along the c axis.

Table S3. Comparison of experimentally identified adsorption sites and calculated binding energies.

		BUT-53	BUT-54	BUT-55	BUT-56	BUT-57	BUT-58
Accessibility of single crystals of guest-adsorbed phase		√	√	√	×	×	√
Adsorption site<br>(C-H…π in blue, C-H…N in pink, and π…π in green)	Site I	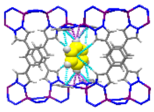	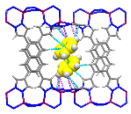	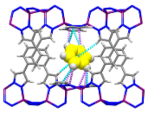	–	–	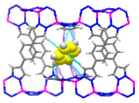
	Site II	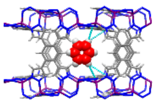	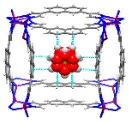	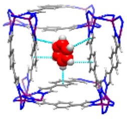	–	–	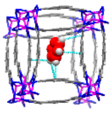
Guest packing		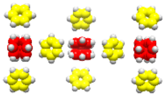	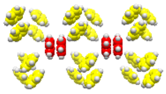	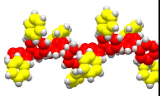	–	–	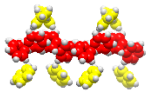
Binding energy	Site I	−116.72	−82.15	−110.06	–	–	−113.91
	Site II	−78.55	−94.68	−72.99	–	–	−109.93
Interaction type<br>(B represents the guest benzene molecules and L represents the pyrazolate ligands)	Site I	C-H(B)…π(L)<br>C-H(L)…π(B)<br>C-H(B)…N(L)	C-H(B)…π(L)<br>C-H(L)…π(B)<br>C-H(B)…N(L)<br>π(B)…π(B)	C-H(B)…π(L)<br>C-H(L)…π(B)<br>C-H(B)…N(L)	–	–	C-H(B)…π(L)<br>C-H(L)…π(B)<br>C-H(B)…N(L)
	Site II	C-H(B)…π(L)	C-H(B)…π(L)<br>π(B)…π(B)	C-H(B)…π(L)	–	–	C-H(B)…π(L)
Interaction distance (Å)	Site I	2.85-3.78	2.63-3.60 for C-H…X;<br>3.66 for π(B)…π(B)	2.89-3.58	–	–	2.82-3.66
	Site II	3.56-3.70	3.11 for C-H(B)…π(L);<br>2.40 for π(B)…π(B)	2.74-3.88	–	–	2.99-3.77

Table S4. Controlled synthesis of supramolecular isomers Co(BDP) and BUT-55, and Zn(BDP) and BUT-58.

	Solvent (mL)	Temp. (^{o}C)	Time (h)
Co(BDP)	10: 2	130	12
BUT-55	8: 4	150	12
Zn(BDP)	10: 2	80	6
BUT-58	10: 7	100	6

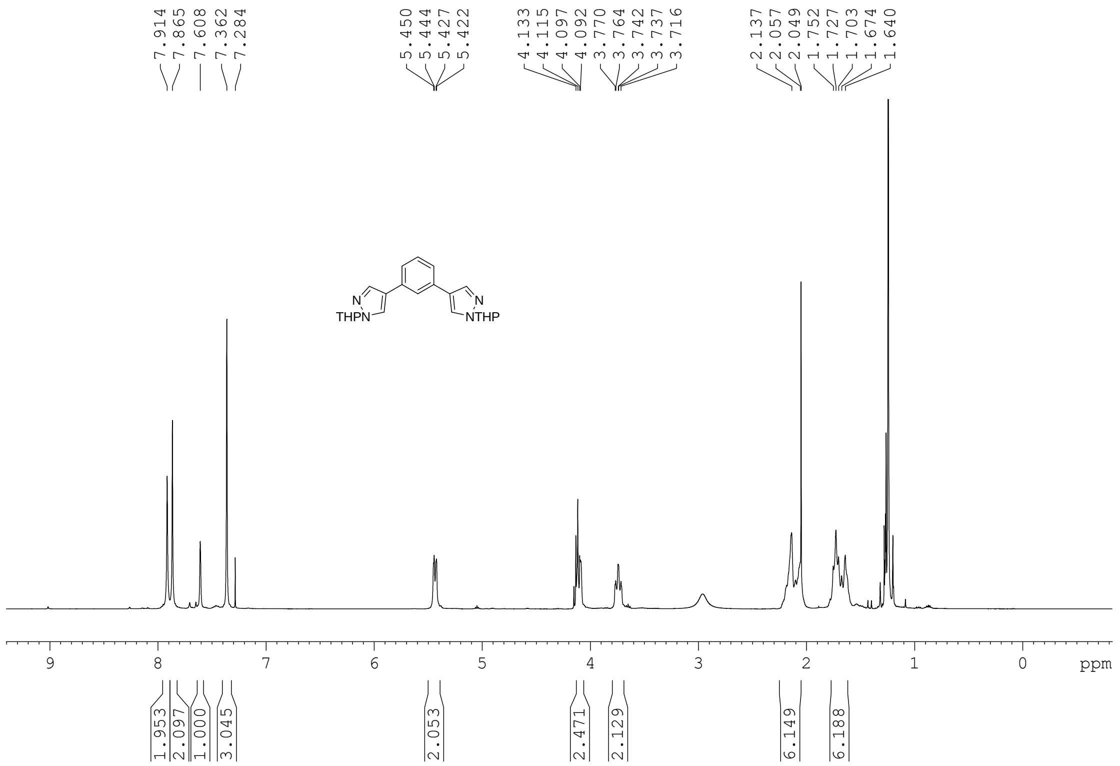

Fig. S45. ^{1}H NMR spectrum of DPB-THP.

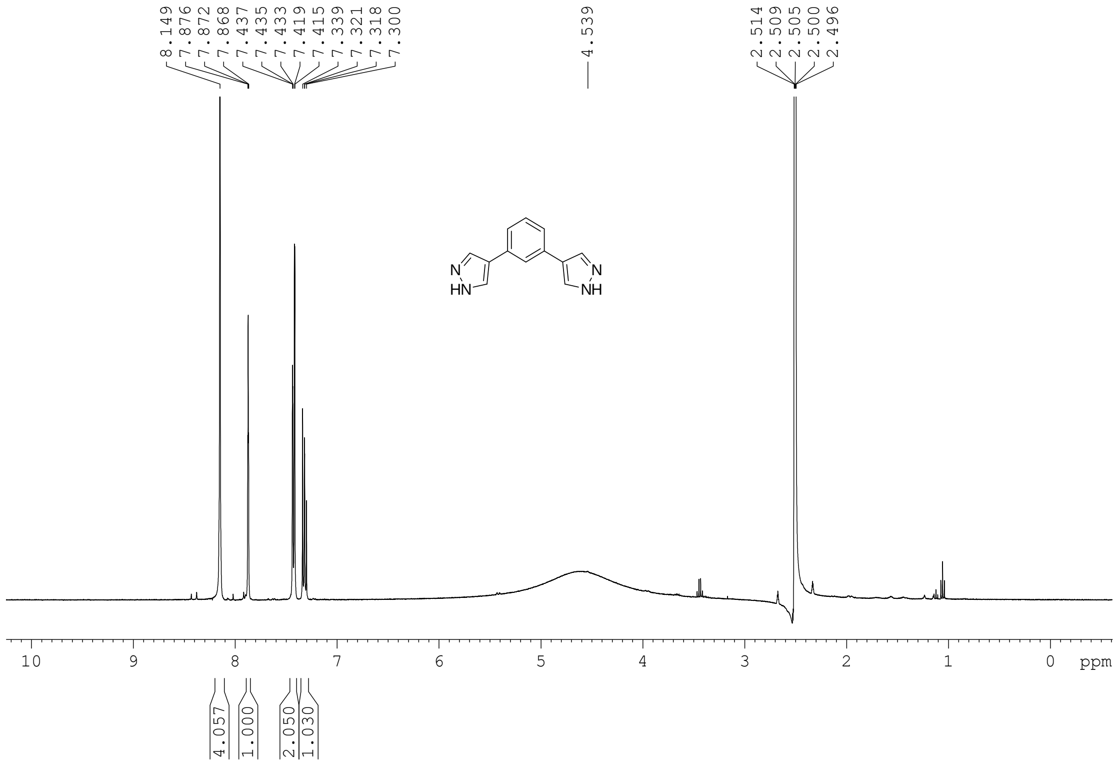

Fig. S46. ^{1}H NMR spectrum of H_2DPB.

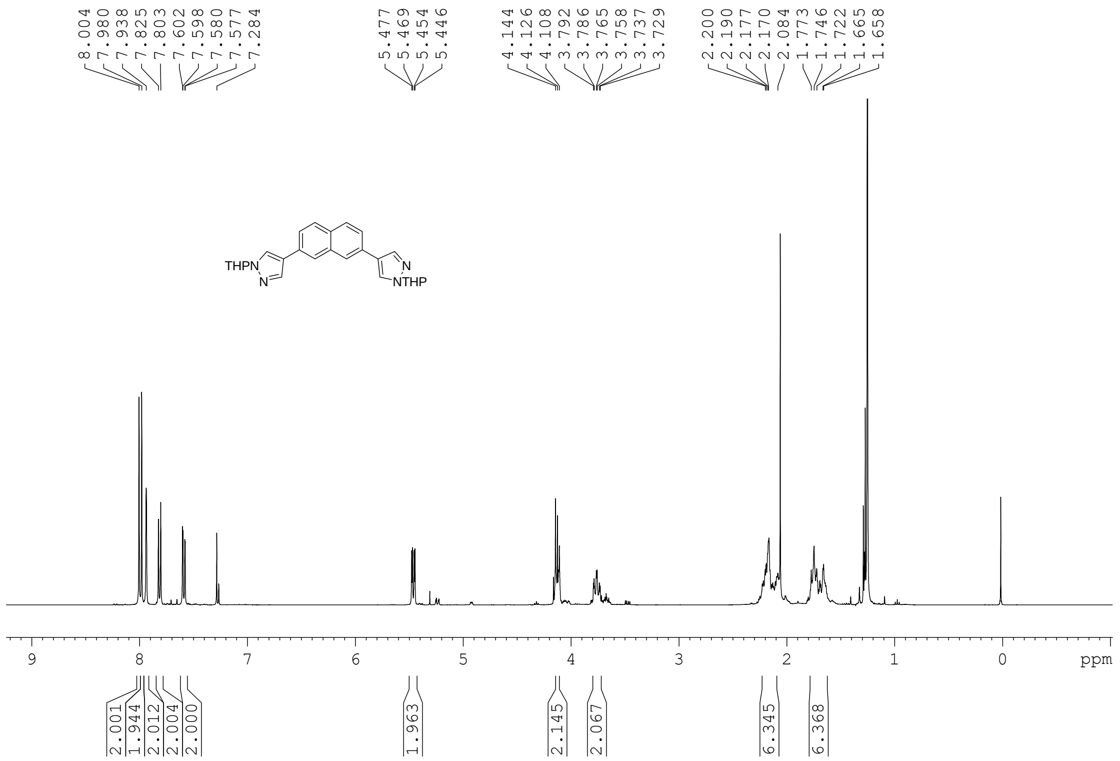

Fig. S47. ^{1}H NMR spectrum of DPN-THP.

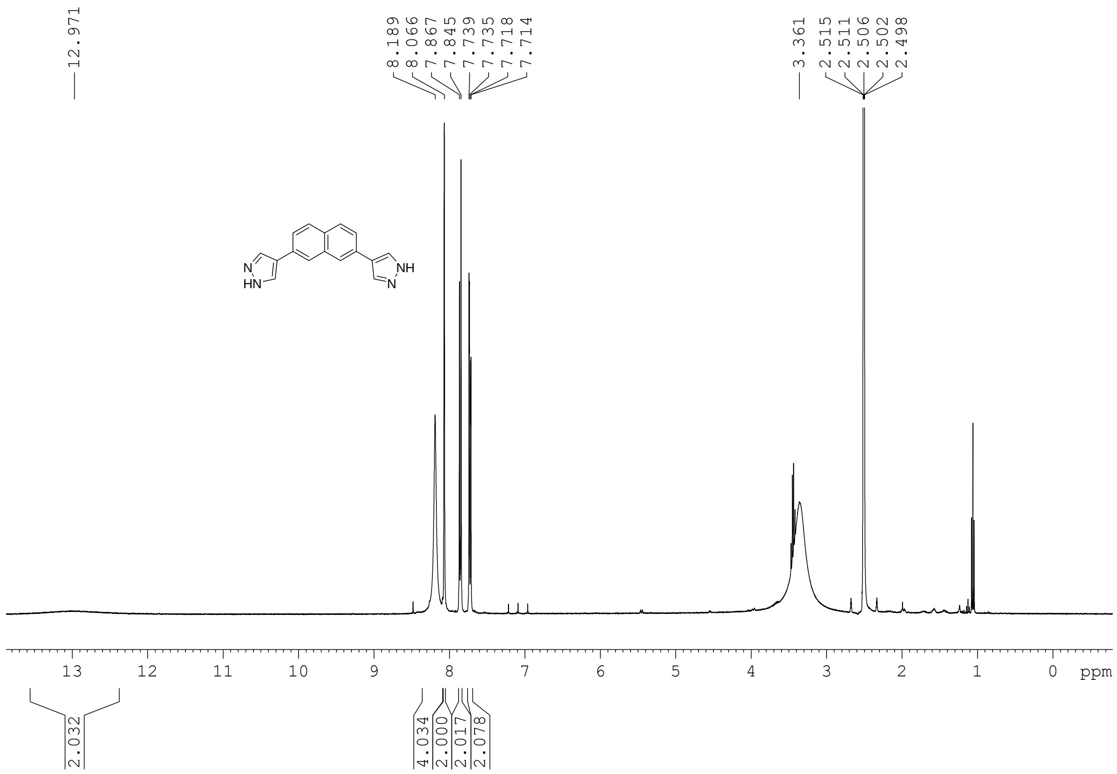

Fig. S48. ^{1}H NMR spectrum of H_2DPN.

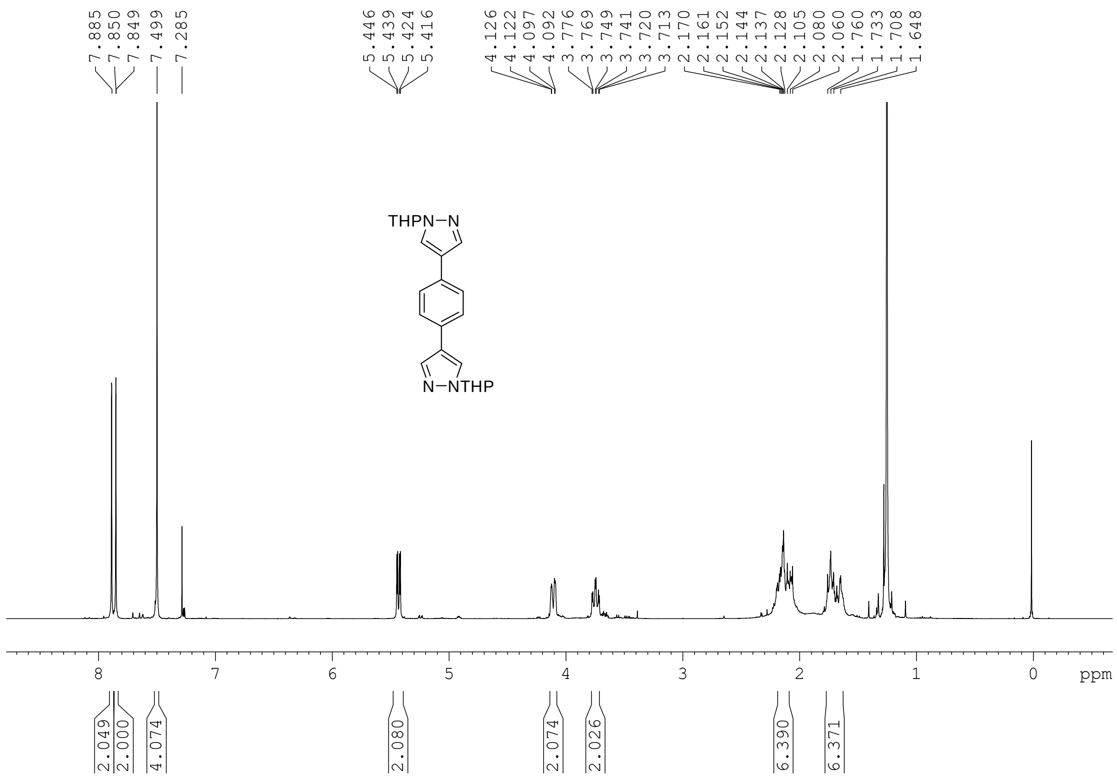

Fig. S49. ^{1}H NMR spectrum of BDP-THP.

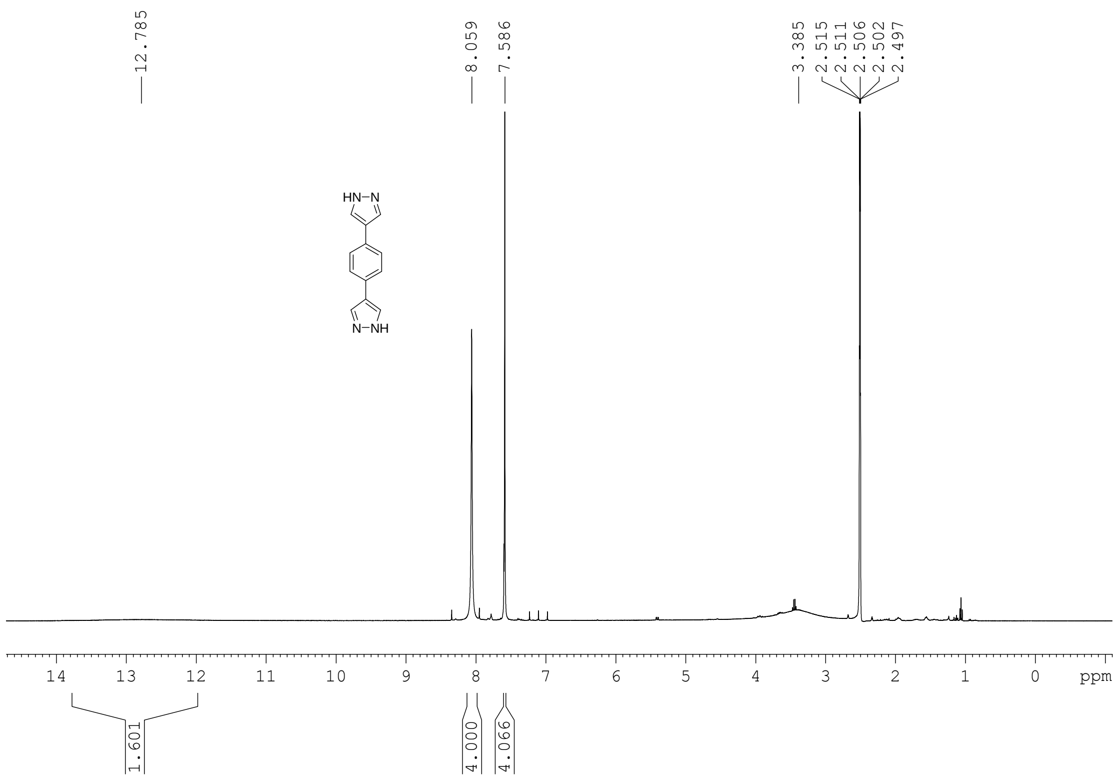

Fig. S50. ^{1}H NMR spectrum of H_2BDP.

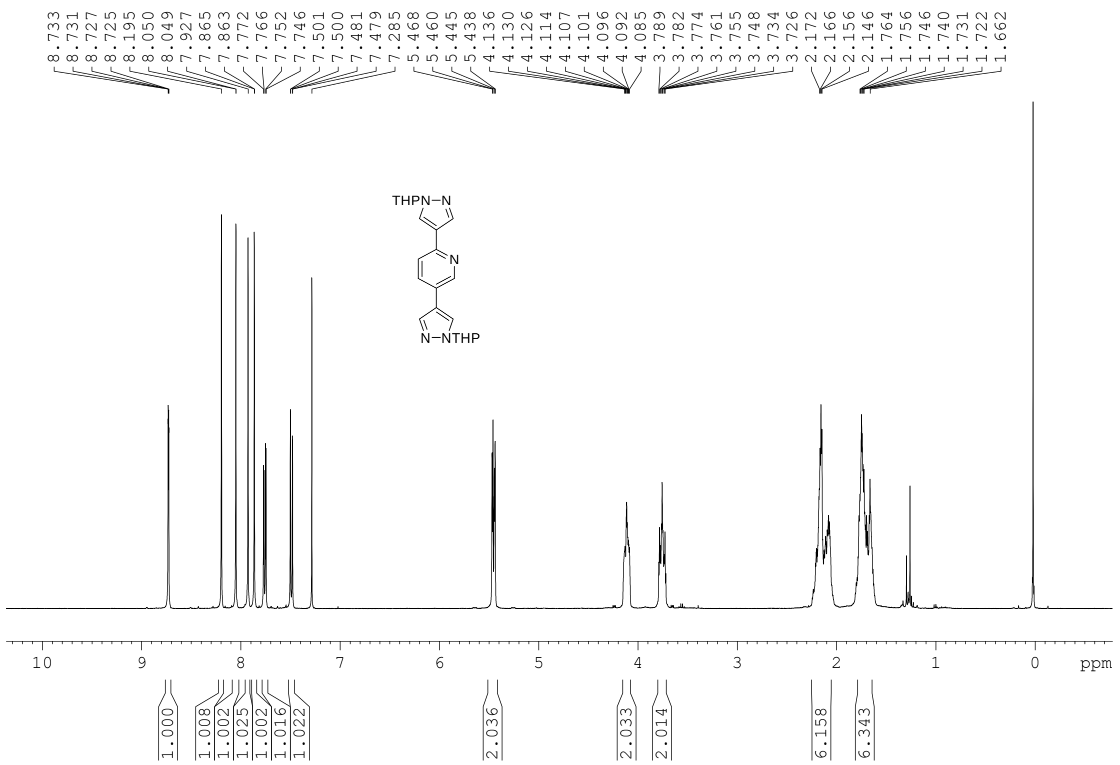

Fig. S51. ^{1}H NMR spectrum of DPP-THP.

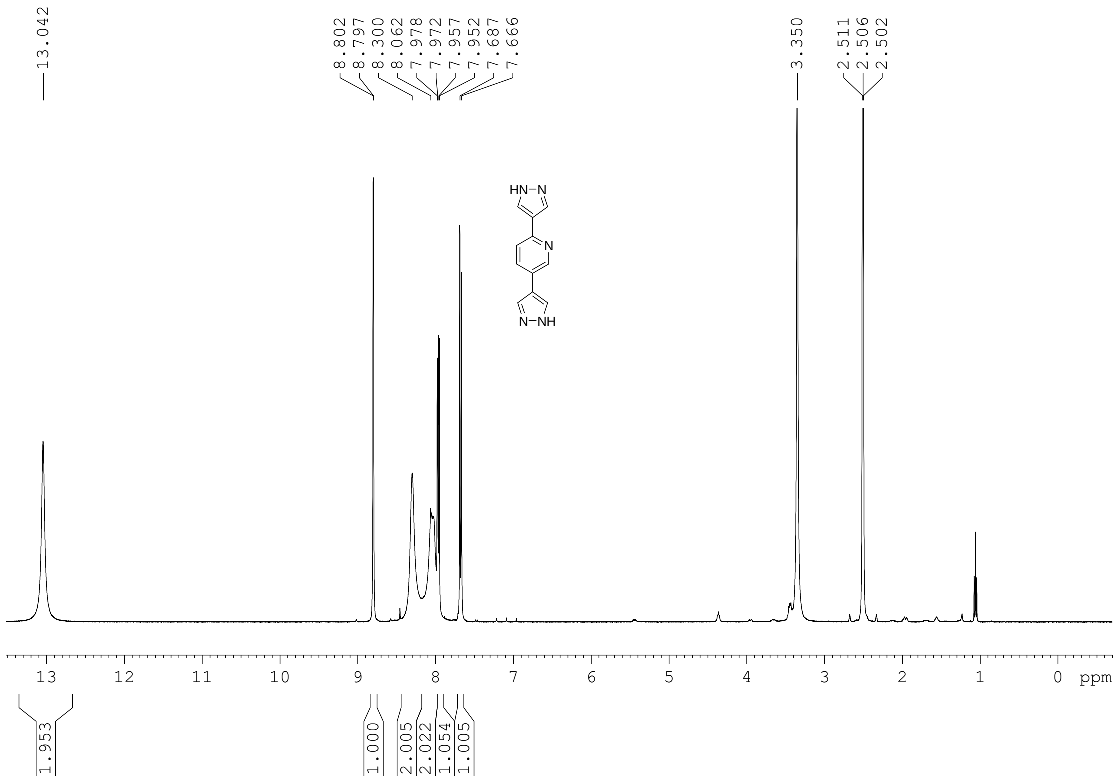

Fig. S52. ^{1}H NMR spectrum of H_2DPP.

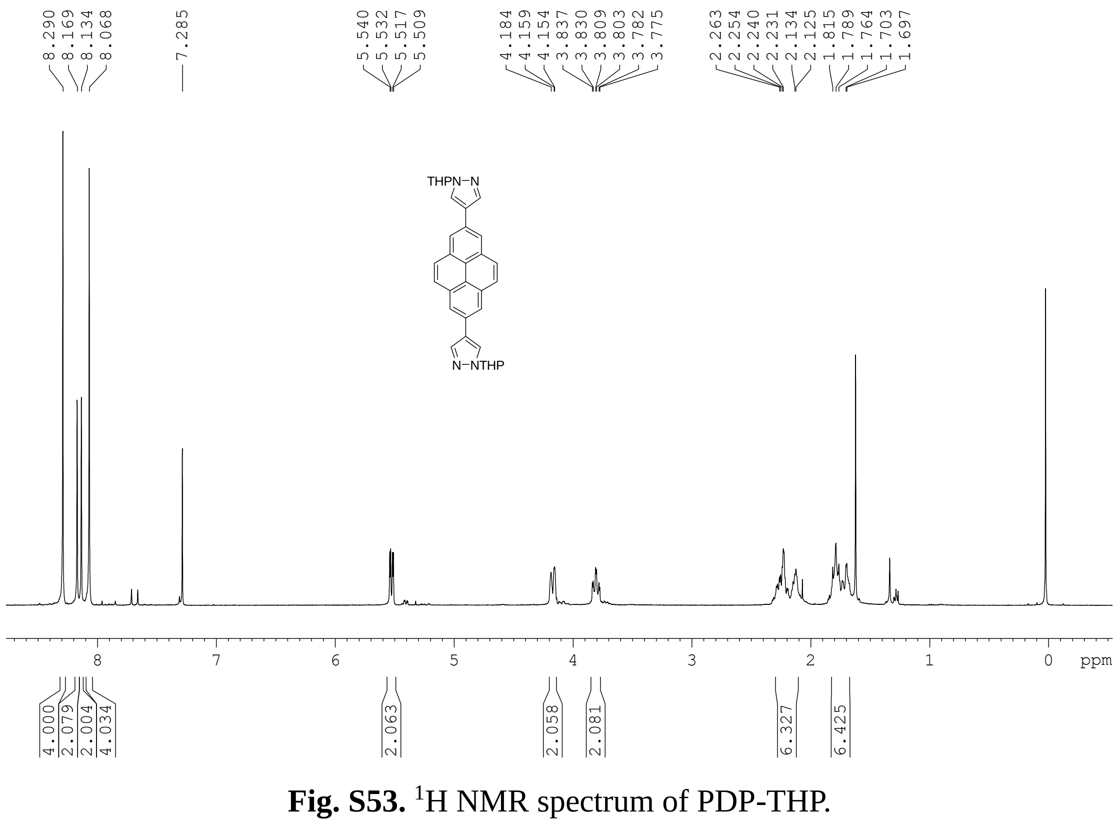

Fig. S53. ^{1}H NMR spectrum of PDP-THP.

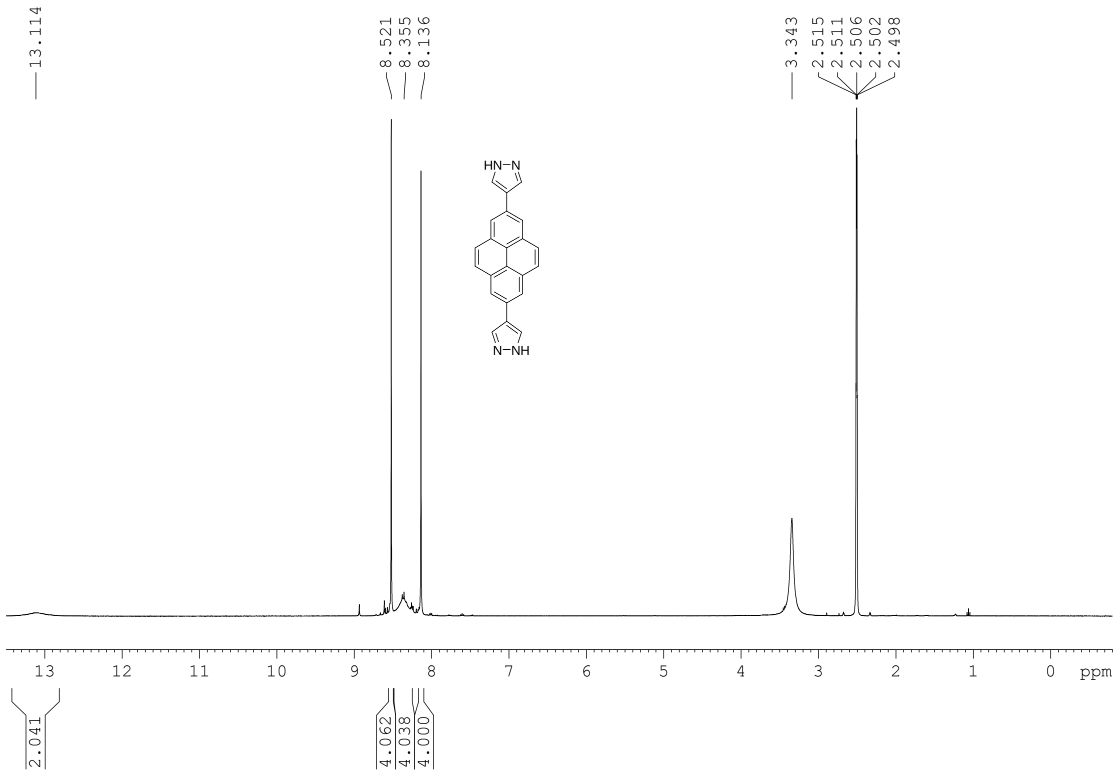

Fig. S54. ^{1}H NMR spectrum of H_2PDP.

Section 9. References

[S1] He, T.; Huang, Z.; Yuan, S.; Lv, X.-L.; Kong, X.-J.; Zou, X.; Zhou, H.-C.; Li, J.-R. Kinetically Controlled Reticular Assembly of a Chemically Stable Mesoporous Ni(II)-Pyrazolate Metal–Organic Framework. *J. Am. Chem. Soc.* **2020**, *142*, 13491.

[S2] Oxford Diffraction. CrysAlis Pro Software, Ver. 1.171.34 (Oxford Diffraction Ltd), **2010**.

[S3] Sheldrick, G. M. A short history of SHELX. *Acta Crystallogr. A* **2008**, *64*, 112.

[S4] Spek, A.L. Structure validation in chemical crystallography. *Acta Crystallogr. D Biol. Crystallogr.* **2009**, *65*, 148.

[S5] VandeVondele, J.; Krack, M.; Mohamed, F.; Parrinello, M.; Chassaing, T.; Hutter, J. Quickstep: Fast and accurate density functional calculations using a mixed Gaussian and plane waves approach. *Comput. Phys. Commun.* **2005**, *167,* 103-128.

[S6] Goedecker, S.; Teter, M. & Hutter, J. Separable dual-space Gaussian pseudopotentials. *Phys. Rev. B* **1996**, *54*, 1703-1710.

[S7] Nie, X., Esopi, M. R., Janik, M. J. & Asthagiri, A. Selectivity of CO_2 reduction on copper electrodes: the role of the kinetics of elementary steps. *Angew. Chem. Int. Ed.* **2013**, *125*, 2519-2522.

[S8] Krack, M. & Parrinello, M. All-electron ab-initio molecular dynamics. *Phys. Chem. Chem. Phys.* **2000**, *2*, 2105-2112.

[S9] Capdevila-Cortada, M. & Lopez, N. Entropic contributions enhance polarity compensation for CeO_2(100) surfaces. *Nat. Mater*. **2017**, *16*, 328-334.

[S10] Clayborne, A., Chun, H. J., Rankin, R. B. & Greeley, J. Elucidation of pathways for NO electroreduction on Pt (111) from first principles. *Angew. Chem. Int. Ed.* **2015**, *127*, 8373-8376.

[S11] Perdew, J. P., Burke, K. & Ernzerhof, M. Generalized gradient approximation made simple. *Phys. Rev. Lett.* **1996**, *77*, 3865-3868.

[S12] Grimme, S., Antony, J., Ehrlich, S. & Krieg, H. A consistent and accurate ab initio parametrization of density functional dispersion correction (DFT-D) for the 94 elements H-Pu. *J. Chem. Phys.* **2010**, *132*, 154104.
